# Multi-Origin GIS Routing for Supply Chains and Logistics in Public Agencies, Municipalities, and Construction 4.0

**DOI:** 10.3390/s26196275

**Published:** 2026-10-03

**Authors:** Pavel Alexandrovich Popov, Hamed Fathnejat, Farzad Jalaei, Arash Hosseini Gourabpasi

**Affiliations:** National Research Council of Canada, 1200 Montreal Rd, Ottawa, ON K1A 0R6, Canada; pavel.popov@nrc-cnrc.gc.ca (P.A.P.); hamed.fathnejat@nrc-cnrc.gc.ca (H.F.); farzad.jalaei@nrc-cnrc.gc.ca (F.J.)

**Keywords:** multi-origin routing, GIS, transportation logistics, decision support, BIM-assisted GIS, public asset management

## Abstract

A Multi-Origin GIS Routing Web Tool is presented with the goal of facilitating GIS analytics for supply chain and logistics decision support for built assets. The tool routes multiple origins to a given destination, plots routes, and calculates travel distances. The tool is easy to use for non-experts, and it is free to use within limits and open source. The tool comes in two forms, including a highly portable web application with a GIS interface and a programmable API that can be integrated into existing programming, analytics, and optimization environments. The Multi-Origin GIS routing tool is built on existing open-source technologies in web-app mapping and GIS analysis, and makes them accessible for non-expert users who do not have the required software development or GIS domain knowledge. The tool provides a higher level of spatial analytics than other web-based tools such as Google Maps, MapQuest, or Bing Maps, without any of the complexity and domain knowledge required to use GIS software such as ArcGIS or QGIS. The tool allows public agencies and municipalities to use GIS analytics to resolve supply chain and logistics challenges while conserving public funds and remaining market-impartial. A case study is presented to show how cost vs. distance traveled analytics can be used for decision support in an AEC project using this tool. The case study results showed optimal supplier choices in terms of both distance and cost, with the trade-offs being clearly visible should a balanced solution be desired. The resulting routing is identical to routing that can be obtained through GIS software; however, the degree of difficulty is much lower, the approach does not require layer data preparation, and the results are summarily presented in an intuitive and easy-to-interpret manner that does not require in-depth examination. In addition, the tool is free for general use cases, which, compared to commercial logistics or research-oriented software, makes it highly attractive to organizations and projects where digitization is at low levels and budgets are limited. This tool represents an important first step in BIM-GIS integration in the low digitization environment of North American AEC and public asset management. Further development will be required to achieve bidirectional information exchange between BIM and GIS, IoT integration, and digital twin functionality. IoT sensor data incorporation, in particular, will enable full two-way data exchange between digital models and physical assets and enable digital twin functionality for logistics operations.

## 1. Introduction

Supply chains and transportation are a large part of infrastructure projects, built asset projects, commercial goods delivery, recycling and rehabilitation projects, and government services. Supply chains are a large part of the costs and carbon emissions of these projects. Digital technologies, specifically Geographic Information Systems (GISs), present an opportunity to improve supply chains and transportation strategies to increase productivity by reducing costs and to reduce environmental impact by decreasing carbon emissions. GIS technologies can provide routing, localization, overlay, multi-destination delivery, and, most importantly, visualization of different, incongruous, complex, and large-scale geographical situation information. These technologies can be used to find optimal routes, predict demand, and situate new facilities to address needs within municipal, provincial, and national jurisdictions.

Governing municipalities, government agencies, and government departments need GIS tools in order to organize and optimize their supply chains and keep track of information coming from multiple sources. Specifically, data on distances traveled is needed to optimize said supply chains. Cost and impact assessments for Architecture, Engineering, and Construction (AEC) projects have traditionally assumed fixed-length travel distances, which is quite inaccurate. In moving towards Building Information Modeling (BIM) and Construction 4.0, GIS technologies fulfill the need for geolocation. Distance calculation and routing can be performed between assets and supply points that deliver materials and services to allow for logistics decision support and the evaluation of environmental impact. GIS technologies can situate BIM models within a mapped representation of physical regions, which allows the coordination and improvement of supply chains to improve productivity and sustainability. In addition, the various elements of projects can be tracked and accounted for, which aids in both planning, adaptability, and accountability.

There is, however, the challenge that GIS technologies can be both costly and challenging to use or incorporate into existing systems. To this end, this work presents a GIS component called the Multi-Origin GIS Routing Webtool. The tool can solve the challenge of routing from multiple source sites to one destination site. This router can calculate travel distances, can visualize the optimal routes, and it can be easily incorporated into existing web applications or programming environments because of its Docker container format. The format addresses issues of compatibility and facilitates use. The router can be queried in large database-style projects with its programmable Application Programming Interface (API). This router is also open-source, which can aid in reducing costs and not showing preferential economic treatment of one private software company over others, which is often an important aspect when stewarding public funds.

This tool is built upon existing open-source solutions, specifically OpenRouteService and Leaflet (version 2.0.0). The mechanisms upon which this tool functions are well known among software developers who work with GIS. However, the packaging of these mechanisms into streamlined web app mapping tools with multi-origin routing, which have the same accessible user interface as Google Maps, has not yet been performed. Though these mechanisms exist and are well known, when they are required for supply chain analytics, they are not accessible without significant software development skills and domain knowledge of GIS. The novelty of the tool lies in the packaging and presentation of existing open-source mechanisms into an accessible tool for non-experts in low-digitization environments for GIS routing analytics.

The presented GIS router for logistics in built asset projects delivers easy-to-use, easy-to-integrate, and free-to-use GIS routing services to government municipalities and departments, such that they can take a practical step forward toward leveraging GIS routing technologies to improve their supply chains and transportation projects. Furthermore, this tool will enable the creation and improvement of supply chains for BIM projects, and can be further developed to provide decision support functionality such that users and planners can ascertain the impact on productivity and sustainability of different logistical decisions.

This GIS routing tool allows for the comparison of different sourcing options via travel distances and cost comparisons. Travel distances offer a practical basis upon which sustainability can be evaluated. Using this tool is an important and practical first step to achieving meaningful BIM-GIS integration. BIM-GIS integration can leverage the benefits of inventory and visualization of built assets of BIM with the geolocation capabilities of GIS to allow more organized, productive, and efficient planning, monitoring, building, maintenance, and demolition of built assets. Research in the area has shown different approaches to achieving BIM-GIS integration, which indicates that the manner of integration is, and will continue to be, industry-, agency-, and situation-dependent. This GIS routing tool, with its flexibility, ease of use, and useful routing analytics, will allow municipalities, governments, government agencies, and other AEC and logistics practitioners to explore leveraging the benefits of GIS routing. The routing that can be performed with the tool is free to a practical scale of data demand. Furthermore, the tool will offer an opportunity to gradually build upon GIS capacity to leverage further benefits of GIS and BIM in projects and organizations. The tool’s GIS capabilities can be enhanced with IoT sensors, which offer a practical pathway to achieve two-way communication between BIM models and geolocated physical material assets, which is the key component for full BIM-GIS integration.

Construction 4.0 encompasses not only fully developed digital twins and real-time cyber-physical systems, but also the progressive digitalization and integration of construction information, analytical tools, and decision-support processes. Accordingly, BIM–GIS integration may occur at different levels of maturity, ranging from unidirectional BIM-assisted GIS analysis to bidirectional and dynamically synchronized systems. The present work represents an initial level of this integration: BIM-related project information and model location provide the construction context, while GIS supplies geocoding, routing, visualization, and supplier-comparison capabilities. The API-enabled and containerized architecture also permits the routing tool to be incorporated into broader digital construction and simulation–optimization workflows. The current implementation constitutes a practical Construction 4.0 component and a foundation upon which two-way BIM–GIS synchronization, real-time sensor updates, or digital-twin functionality can be developed.

The study demonstrates a BIM-assisted, API-enabled GIS logistics workflow that represents an incremental and practically deployable form of Construction 4.0 integration in the form of the Multi-Origin GIS Routing tool. The advantages of the Multi-Origin GIS Routing tool can be summarized as follows:

- The tool performs multi-origin routing, which is not natively available in other web-based GIS tools.
- The tool allows for options regarding cargo type and transport vehicle dimensions to ensure that selected routes fit transportation needs.
- The web-app format makes the tool easy to install and use on a large variety of computer platforms.
- The routing tool does not require the costs of commercial GIS analytics or logistics software.
- The tool does not require data preprocessing or domain knowledge that is required to use GIS spatial analytics software.
- Free to use and a simple, intuitive user interface (UI) make it easy for non-expert users to use within low-digitization organizations or work environments.
- Results are presented in a concise, intuitive manner.
- No other tool has multi-origin routing analytics and is easy to use for non-experts.

The rest of this paper is organized as follows: Section 2 describes the literature relating to current research activities in using GIS for transportation and logistics applications. It examines the challenges and shortcomings of current routing-based GIS solutions. Section 3 describes the methodology of the proposed GIS routing tool. Section 4 presents a case study demonstrating how the tool can be used to compare procurement costs vs. distances traveled for a model AEC project. This section also compares the tool to other multi-origin routing approaches. Section 5 discusses the results of the case study and how the tool addresses the current needs of the public sector for asset management and logistics. Additionally, how the tool can be used for decision support for AEC projects is also discussed. The discussion also includes in-depth explanations of limitations and potential directions for further research and improvement. Section 6 concludes the paper and includes a brief discussion on future applications.

The Multi-Origin GIS Routing web tool was built with the intent of aiding the research community. Access to the tool may be obtained upon reasonable request. Interested users are encouraged to contact the authors for access to the tool.

## 2. Literature Review

There has been significant interest in using GIS for solving supply chain and transportation logistics challenges, especially among municipalities and governments.

Research has delved into a wide range of applications from cargo delivery to supply chain management and planning. Some noteworthy applications of GIS for transportation logistics include [1], where GIS was used to analyze the susceptibility of a road network to hydro-meteorological disasters, with a focus on the road network links used for vaccine distribution. The supply chain for electric vehicle (EV) batteries is explored in Ref. [2] to identify optimal locations in a study area for battery pack dismantling hubs and quantify environmental and economic impacts of battery pack recycling for EVs. The framework presented in Ref. [3] optimizes transportation routes of biomass for biogas energy production while applying GHG limits, and selects sites for biogas plants. GIS analytics are used to examine a municipal solid waste (MSW) supply chain in Ref. [4], which includes identifying suitable sites for transfer stations, optimizing transport routes for MSW, and predicting the life expectancy of existing landfills with waste valorization for enhancing the circularity of MSW in a city. The work in Ref. [5] evaluates the impact of a potential logistics center for delivering goods based on economic, social, and environmental factors by using GIS and Strengths, Weaknesses, Opportunities, and Threats analysis (SWOT), and applies limitations and considerations such as transport management, infrastructure, fleet attributes, and logistics and market features. The work of Ref. [6] proposes using photovoltaic (PV) panels to supply the electricity needed for replacing diesel school buses with electric school buses. The study analyzed school data to identify which schools could generate solar energy to meet the energy demands of their busing needs, and how much space these schools would need for PV equipment, based on geography.

Some additional works that deal specifically with approaches that are applicable or directly designed for public infrastructure, building, and transportation services projects include Refs. [7,8,9,10,11], which deal with urban and interurban transportation infrastructure; Ref. [12], which uses global positioning system (GPS) data for freight monitoring; and Refs. [13,14,15,16,17], which examine site selection for bioenergy plants, with Ref. [18] specifically focusing on reducing the environmental impact of bioenergy supply chains. Additionally, Refs. [19,20] incorporate GIS data into Life Cycle Analysis (LCA) for their BIM-enhanced building projects. The relationship between building stock and transportation stock is examined in Ref. [21] for climate impact mitigation. Further applications relate to waste collection [22,23], waste biomass potential for energy generation [24], and centralized vs. distributed biomass processing [25]. The studies [26,27] are concerned with hydrogen production. Goods delivery is another useful application, and it is the focus of Refs. [28,29,30] for consumer goods. Public education exam book delivery is examined in Ref. [31]. Resource collection is examined in Ref. [32] for dairy supply chains, Ref. [33] for aggregate rock source identification, Ref. [34] for coastal CO2 storage and enhanced oil recovery, Ref. [35] for crude oil transport monitoring, and for water fisheries in Ref. [36]. HAZMAT transportation management is the focus of Refs. [37,38]. A review of how GIS technologies can be further applied for asset management based on asset classes can be found in Ref. [39].

GIS technologies as they relate to transportation and logistics, specifically on the data processing side, can be separated broadly into several categories: overlay, routing, multi-destination pickup and/or delivery, network analytics specifically related to affected areas, flow and capacity, and optimal location selection. While many approaches exist on the sensor and data collection side of things, which is the process of using different sensors and data collection strategies to collect and produce the needed GIS data, examining these strategies in significant detail is beyond the scope of this paper.

Overlay involves taking different information layers and seeing where overlap occurs. This process uses either rasterized or vectorized data and, in one shape or form, is applied on some level in the vast majority of GIS processes, technologies, and analytics. In the work of Ref. [40], traffic congestion levels on roadways are evaluated using convolutional neural network (CNN) models, and the related congestion classifications are overlaid onto a road network. The study in Ref. [6] is built principally on overlay, and it analyzes the electrical generation potential of PV installations on school grounds based on geography. The geographic data on sunlight received is overlaid onto school location and land area data to determine the electrical generation potential of schools in order to power electric school buses. Overlay of GIS data is used in Ref. [41] to place potential road hazards from different sources onto a road network for delivery vehicle routing. The study creates supply logistics software that allows for more accurate delivery time estimates. A number of additional works make specific mention of overlay-related approaches, including Refs. [8,9,10,14,16,18,20,21,26,27,29,33,35,37,38,42,43]. These works can be consulted for further insights into overlay applications.

GIS network models can be used with shortest path finding algorithms to find optimal routes, and this is the basis of many current GIS applications where optimal routes improve productivity and sustainability. In Refs. [32,36], pathfinding is used for food resource supply chains. Waste disposal routing as part of LCA is used in Ref. [20]. Shortest path finding is used in Refs. [15,17,18,24] for biomass delivery for energy generation purposes. In Refs. [26,27], it is used in hydrogen delivery supply chains. Flight paths are found for drones for goods delivery in Ref. [28]. An interesting example is the work of Ref. [44], which optimizes planning of transit routes and power lines using topography represented by a weighted grid surface model and Dijkstra’s algorithm. Routing is used in Ref. [4] to optimize transport routes for municipal solid waste in their analysis of waste management and landfill life expectancy. GIS data is used to optimize for air traffic in Ref. [45], which results in finding non-overlapping shortest paths with safety margins for commercial airliners and other aircraft. Shortest path analysis is also used in Ref. [42] to optimize biomass delivery to power plants under different use cases and availability scenarios. Additional examples of shortest path finding can be found in Refs. [2,3,11,12,35,37,38,41,43,46].

A related common application of GIS is finding routes for multiple destination pickup and/or delivery situations. These problems and their solutions are academically referred to as traveling salesman problems. These problems are computationally prohibitive to solve for optimal solutions and are instead approximated with heuristic algorithms that yield approximations of the optimal solutions. Many GIS research works use multi-destination pickup/delivery planning to improve transport and logistics strategies. A multi-destination vehicle routing problem is solved in Ref. [23] with limited capacity and time windows, in order to plan the collection of vegetable oil waste in an urban area. The study in Ref. [31] uses multi-destination pickup/delivery planning using network analysis in GIS to formulate a delivery plan for exam booklets for secondary education standardized testing with their fleet of transport trucks. The delivery plan has a lower combined travel distance than the baseline. Further examples of multi-destination pickup and delivery applications can be seen in Refs. [5,13,22,30,36].

GIS network models can be further applied to solve more complex problems using more complex network analytics. Network analytics are used to calculate affected areas of studied geographical entities and to perform flow and capacity analysis. Affected areas, in essence, are an ensemble of shortest path routes from a certain origin to any reachable destination given certain distance, time, or calculated energy limits. Affected area analysis is used in Refs. [13,14,15,16,17,21,25,32,34] in a range of applications. The work of Ref. [1] uses network analytics to assess areas affected by flooding. Accessibility of a dense urban area is evaluated in Ref. [7] using an isochrone model, which, in its application, evaluates the reach of a person traveling within the urban area, modeled by their GIS network, from given origin points. The area of human exposure risk from liquid petroleum gas transportation is evaluated in Ref. [37], and the routing is optimized to reduce the risk. The work in Ref. [8] likewise uses a road network to assess the driving coverage of electrical charging facilities located at truck stops along the US highway network.

Building upon affected area analysis, flow and capacity analyses are calculated by performing many affected area analyses from multiple origins in a network simultaneously, and keeping track of how much proportional connectedness each entity in the network receives from each of the considered origins. This proportional connectedness is a representative fraction of how much substance or material is delivered to an entity. To use the example of a hydraulic network, each origin is a source of water, and each entity of the network is a junction that delivers water to users. The proportional connectedness is an analog for the amount of water that each entity receives. Flow and capacity of a railway and truck cargo freight system were estimated in Ref. [46], in their coupled simulation optimization framework, to determine where bottlenecks lie and the impacts of investing in supply chain improvements relative to greenhouse gas (GHG) emissions. Anonymized GPS data of freight trucks is used in Ref. [43] to extract delivery paths and analyze the ensemble of these paths to forecast freight shipping. Environmental impact is studied in Ref. [19], where transport between multiple points of interest and residential building projects in a dense urban area is examined to determine the effect of residential dwelling densification. Railway and highway resilience for military transport is evaluated in Ref. [11] by determining the effect of disruptions on transportation from military installations to airports and seaports. Flow and capacity analysis is also used in Refs. [9,12,22,35,38,40,47], with a dataset suggested for flow and capacity modeling applications.

Suitable location analysis is a useful outcome of combining these network analyses with overlay. This is also called location–allocation. The network model is evaluated to find entities that have high connectivity or that connect to the greatest number of substance or material origins, and these are constrained by limiting spatial factors and desired geographical factors, which yield optimal locations. These analyses can be found in Refs. [4,13,14,15,16,17,24,25,27,33,36,48]. The work of Ref. [3] examines site location for potential biogas plants in order to build a recycled biomass energy production network. Site locations for battery pack recycling are determined in Ref. [2], which is a necessary step in supporting electric vehicle usage. Optimal locations for charging stations for delivery drones are found in the work of Ref. [28] for case study cities. Similarly, the authors of [29] find locations for package distribution centers for a last-mile cargo-bike delivery network. Suitable sites are located in Ref. [49] for sustainable aviation fuel production based on reachable raw material areas found via affected area analysis. It is interesting to note that all these GIS analytics approaches build upon each other, or are otherwise interconnected.

The applications of logistics projects that use GIS vary significantly between built asset-related projects, accessibility, and goods delivery. As such, GIS logistics tools have applications in AEC, public asset management, industry support, and commercial shipping. Built asset projects are of particular interest to governments and government agencies because these projects often have an impact on infrastructure and also have high environmental impacts. A very good example of a built asset project is the work of Ref. [8], which is not only about good delivery but about the infrastructure that is needed to support it in a green energy transition. The research determines where electric fast-charging stations will need to be built in the USA highway network to provide sufficient electric charge coverage for cargo freight electric trucks in order to replace diesel trucks and achieve sufficient transport range within the highway network. Another interesting work is Ref. [20], which combines BIM with GIS in order to optimize the construction design, material selection, and transport, operations, maintenance, and waste management of a built asset. Results show a significant reduction in GHG emissions in the materialization, operation, and demolition stages of a building’s management. BIM and GIS are used in Ref. [41] in a digital twin framework for real-time logistics. The framework can predict arrival times and logistical risk of different building components using BIM, GIS, and Internet of Things (IoT) sensors. The digital twin updates virtual assets based on a BIM model and IoT sensors, and the virtual asset’s components are transferred to GIS software for routing with the imposed logistical limitations of the given building components. GIS logistics technologies can help governments, government agencies, and private-sector AEC practitioners track spending, plan projects more efficiently, and weigh the outcomes between different strategies, and there is considerable interest in researching and applying these applications.

A little over half of the above-mentioned methods rely on commercial software, and there are inherent limitations of using commercial software, not the least of which is the cost of use. Municipalities and government agencies usually face budget limitations on asset maintenance and logistics projects. Having free software would be an asset for keeping costs down. Government agencies are supposed to serve the public good and, as such, should not show economically preferential treatment to a private enterprise over other enterprises in a given service field. Commercial software also tends to be less flexible than open-source software, which can be more easily modified and adapted for use in new use cases and applications. Conversely, open-source software can be quite difficult to install, use, and develop, and these factors are especially pertinent in the fields related to construction where there exists a low level of digitization globally. This low level of digitization is also a likely aspect of government departments and municipalities that have limited budgets and an ever-increasing portfolio of built assets and provided services. How approachable and complete a given GIS solution is will drive adoption among practitioners. Software should be such that a trained professional should not be required in order to use it.

To summarize the research reviewed on GIS tools for logistics, Table 1 mentions the key aspects that have been reviewed and how they relate to the use of GIS tools for municipalities, government agencies, and AEC.

**Table 1:** Summary of applications and technologies for GIS for logistics.

Element	Relationship to Logistics and GIS Routing Tools	Specific Examples
Potential Applications	There is a range of applications that have been researched in the GIS literature with regard to logistics, which represent potential future applications and projects for municipalities, government agencies, and AEC practitioners.	- Disaster susceptibility of transport network [1] - Site selection for battery pack dismantlement hubs [2] - Route optimization for biogas [3], waste [4], and other materials. - Impact assessment of new facilities [5] - PV power generation for EV [6]
Existing Applications	There are existing applications that are already of interest to public authorities and AEC practitioners which involve logistics that can be improved using GIS analytics. These studies are of significant interest to public authorities looking to improve the efficiency and cost-effectiveness of these types of projects within their jurisdictions.	- Cargo Delivery [28,29,30] - Urban transport infrastructure [7,8,9,10,11] - Freight monitoring [12] - GIS and BIM Enhanced Building Projects [19,20,41] - Building stock and transport stock analysis [21] - Biomass energy generation [13,14,15,16,17,18] - Waste collection, Refs. [22,23] - Hydrogen production [26,27] - Government document delivery [31] - Resource collection and Transport [32,33,34,35,36,37,38]
Overlay	This is a core GIS method that takes different layers of geographical information and creates areas and entities of interest. These areas and entities are used for a wide range of logistics analyses for different asset management and AEC applications. The results of the overlay are used to build graphs representing a physical area, which are then processed with routing algorithms to find paths between origins and destinations, among other analyses.	- Traffic congestion [40] - PV power generation for school bus EVs for schools [6] - Routing amidst road hazards for cargo delivery [41]
Shortest Path Routing	Technology that traverses graphs built from overlay operations on GIS data. This technology finds optimal routes between different points and is a core element of interest for route planning, logistics, and impact assessment. Optimal routing can save fuel costs and reduce delivery time, minimize disruption to area operations, and highlight key areas for development and protection.	- Food resource supply chain optimization [32,36] - Waste Disposal [4,20] - Biomass delivery for energy generation [15,17,18,24,42] - Hydrogen energy supply chains [26,27] - Flightpaths for drone delivery [28] and commercial aircraft [45] - Transit route planning [44]
Multi-Destination Routing	A more challenging evolution of shortest path routing that has multiple delivery points along a trajectory and tries to optimize the route to minimize travel-related costs. This has applications in logistics delivery of goods over large areas.	- Multi-site waste vegetable oil collection [23] - Delivery of exam booklets to multiple schools [31]
Network Analysis: Affected Area	Affected areas are an ensemble of shortest path routes from a certain origin to any reachable destination given a certain distance or time. These types of analyses are useful in determining the impact or reachability of new building and infrastructure projects.	- Flooding impact [1] - Accessibility of dense urban area [7] - Human exposure from HAZMAT transport [37] - Driving coverage of EV charging stations [8]
Network Analysis: Capacity and Flow Analysis	Capacity and Flow analysis is, broadly speaking, an ensemble of affected area analyses from multiple origins in an area simultaneously, and keeping track of how much proportional connectedness each entity in the area receives from each of the numerous origins. This has applications in infrastructure and traffic projects.	- Railway and freight cargo capacity analysis of a transport system [46] - Freight forecasting [43] - Environmental impact of urban transport [19] - Resilience of railway and highway transport for military transport [11]
Suitable site selection	A combination of network analysis methods with overlay. Sites are identified that affect or connect to the largest areas based on numerous constraints and desired factors. Has applications in building planning.	- Site selection for biogas plants [3] - Site selection for battery pack recycling hubs for EVs [2] - Drone charging stations for delivery drones [28] - Distribution centers for last-mile cargo delivery [29] - Aviation fuel production sites [49].

A key takeaway from this literature review is that there are existing and potential future applications of using GIS technology for logistics in public service, building, and infrastructure projects, as well as other AEC projects. The majority of these GIS technologies in some way touch upon routing, of which multi-origin routing is a very flexible and useful variant. Existing easy-to-use GIS routing tools such as the Google Maps web-app, MapQuest, and Bing Maps, which may be very attractive to non-experts in low digitization environments, do not natively provide multi-origin routing within the web-apps and have API that may be intimidating or difficult to use. Our streamlined web-app GIS multi-origin routing tool has a streamlined web-app user interface (UI), which is very similar to those web-apps with which users are familiar. It also has an easy-to-use simplified API. The Multi-Origin router gives better routing analytics than Google Maps, MapQuest, and Bing Maps and allows users to implement and explore a major portion of the different analytics approaches used for logistics in the GIS field without the need for costly and complicated GIS analytics software.

Furthermore, a wide variety of GIS applications have been developed for AEC and public asset management that specifically examine and address logistics challenges of supply chains and provide effective solutions. These applications have demonstrated benefits of improved cost productivity, improved quality of service, monitoring, and delivery time, and reduced environmental impact. In short, the current research is progressively addressing the needs of public asset management. However, there are several significant barriers to adoption. These barriers are complex software, reliance on commercial software, and the low digitization environment of many municipal and governmental authorities in the realm of AEC and asset management. There is a need for free and open-source GIS software. It should be easy to use and flexible.

Many GIS approaches are in some ways built on routing, so a routing GIS tool which can be built upon and is easy to use would have a big impact. Further to that point, asset management, as well as BIM, are not singular activities or technologies, but processes. As such, any useful GIS tool should be able to integrate into either process. That is why open source is important, such that any software can be freely integrated into different workflows. A comparison of existing GIS mapping tools is made in Table 10, where these tools are also compared to the proposed Multi-Origin GIS Router. The table indicates the particular differences between existing tools and highlights the needs that are yet to be addressed.

There is a specific need for a free, user-friendly, and open-source routing GIS tool that can integrate into asset management processes and emerging BIM workflows. This is specifically the gap that is addressed by the presented Multi-Origin GIS Router, which can help organizations leverage the benefits of GIS analytics by removing the adoption barriers of complexity, cost, and reliance on commercial software. It can further be built upon as required based on needs and available resources. The details of the Multi-Origin GIS Router will be presented in the next section.

## 3. Methodology

### 3.1. Multi-Origin GIS Routing Webtool

The Multi-Origin GIS Routing Webtool is a lightweight, web-based Geographic Information System (GIS) routing tool designed to compute routes from multiple origins to a single destination. The system provides users with summarized distances and travel times for each origin and automatically identifies and highlights the fastest route among them. These route distances are a first step to performing accurate sustainability assessment and can be used in a broader BIM-GIS logistics decision support system.

Further integration with IoT sensors would allow the tool to update routing information in real-time with changing traffic, routing, and hazard conditions and is a logical next step for development. IoT sensor information from supplier vehicles and construction material installation would establish a channel of communication back to the BIM model of a given project to allow for logistical tracking and improved routing accuracy. The Multi-Origin GIS routing tool with its programmable API is poised for further development and further integration with IoT sensors for bidirectional BIM-GIS information exchange.

The tool supports different transport modes, including passenger vehicles, heavy goods vehicles (HGV), and optional HAZMAT routing for hazardous cargo. Users can select the appropriate profile depending on their needs.

The routing backend is exposed through a set of APIs that wrap OpenRouteService (ORS) [50] endpoints, providing geocoding and routing functionality while keeping the API key secure. OpenRouteService [50] is the library that is used to perform GIS analytics. It is free to use within reasonable use limits, and it is open-source. To ensure portability and reproducibility, the tool is packaged as a Docker container, enabling simple deployment and configuration. The frontend is served as static assets, while the backend runs as a containerized microservice. A reverse proxy handles requests to the endpoints. The tool is built upon existing open-source assets for mapping and multi-origin routing; all of the software technology used is well established and readily available. However, the use of these software technologies requires advanced software development skills and labor in order to tailor these technologies to a given work environment or project. The main innovation of this tool is to package these assets into a Google Maps-style web app, which allows users to use highly useful multi-origin routing analysis without software development skills or domain knowledge of GIS. This enables non-expert users in low-digitization environments to use multi-origin routing GIS analysis to improve and manage supply chains and supplier choice in their asset management and AEC projects.

### 3.2. Workflow

The system architecture consists of two main components:

**Frontend (browser)**: Built using *Leaflet* (v2.0.0) and vanilla *JavaScript*, it provides an interactive map and dynamic user interface for adding and managing origins and destinations. Users can visualize routes, compare distances and travel times, and adjust map views dynamically.

**Backend microservice**: Acts as a secure proxy to the OpenRouteService library and its APIs. It handles geocoding and routing requests, ensuring API credentials remain hidden from client-side code.

Each origin and destination is geocoded using the ORS services (autocomplete, search, or reverse geocode). The frontend sends a routing request for each origin–destination pair via the backend proxy. The returned GeoJSON data is rendered on the map with distinct colors for each route. A results table displays distance and travel time, highlighting the fastest route.

Additionally, when the user selects the *driving-hgv* profile, additional input fields appear for vehicle dimensions, weight, axle load, and hazardous materials. These parameters are passed to the routing engine to avoid roads with restrictions for heavy or hazardous cargo.

Each network call checks for success and provides informative error messages without blocking other routes. Autocomplete requests are debounced to reduce API load. User interface (UI) interactions are optimized to prevent input loss during suggestion selection.

The system overview diagram in Figure 1 illustrates the interaction between the frontend (Leaflet-based map), backend microservice, and ORS APIs. The tool can depict and analyze typical workflows such as multi-origin routing for delivery optimization, HGV route planning with dimensional constraints, and passenger car routing for travel-time comparison.

The tool is distributed as a dockerized web application for ease of setup and portability. Users can deploy it locally or on a cloud server by running the provided Docker configuration. Runtime parameters (e.g., default map center, zoom level, and optional country filters) are configured through an env.js file, which is read at runtime by the frontend. Users who are interested in using the tool and incorporating it into their projects are encouraged to contact the authors with their request.

The Routing Configuration/API logic components can be used with the graphical user interface and with the programmable API. The user interface of the tool can be seen in Figure 2. It is a layout similar to other web-app mapping software such as Google Maps or Bing Maps. The user selects a destination and can add origins as desired. The diagram in Figure 2 displays the result of a simple use case. Additional options are available with different vehicle profiles. These options are a default car profile or a heavy vehicle profile. The heavy vehicle profile tailors the routing to the transport limitations of heavy vehicles and hazardous materials (HAZMAT). Figure 3 below shows the use of the tool with the vehicle profile selector, specifically the heavy vehicle profile. A user can select weight limits and vehicle dimensions, and the routing will take that into account by finding paths that can accommodate the limitations.

The Multi-origin GIS Router can be called using the following function in the software library. The function routes 1 origin to 1 destination. It can be called for as many origins as a user requires. It uses the following function call:

getRoute(origin, destination, profile, hgv)

This function returns a JSON object with the route information. For more information, the software documentation can be consulted.

## 4. Case Study

A case study for a model multi-level mixed-use building project was conducted using the Multi-Origin Routing GIS tool to provide analysis for decision support, specifically for supplier selection for takeoff materials using distance traveled vs. cost analysis. A model building project was used to simulate a BIM workflow using a sample Industry Foundation Classes (IFC) model file for a multi-storey building for visualization, and a list of materials from a material takeoff was generated using the IFC OpenShell library to process the IFC model. Four classes of materials were selected for sourcing estimation. IFC, which stands for Industry Foundation Classes, is an open-source file type for modeling used in BIM workflows. IFC models can be used to generate a bill of materials or material takeoff in a BIM-GIS workflow, and provide the advantages of digital inventory and visualization. The material sourcing costs were then calculated based on known supplier addresses for construction materials in Eastern Canada. These supplier data were also used in [51] where the Multi-Origin GIS router was used as part of a route-aware emissions assessment. The case study presented there is an excellent example of carbon emission calculation. The selected worksite location was also specified in Ref. [51]. The case study presented here focuses instead on supplier selection, which often requires a balance of costs and environmental factors. The flowchart of the simulated BIM workflow of this case study can be seen in Figure 4. This figure shows the major steps of the process, including material takeoff, material costs, and multi-origin routing, as well as distance vs. cost analysis, which will be explained further in the remainder of this section.

This simulated BIM workflow illustrates how the GIS tool can be used to provide decision support and is a proof of concept designed to highlight the type of analytics that can be leveraged by practitioners and organizations, and is only loosely accurate physically and budgetarily. Validation that would be performed in a real project is beyond the scope and does not serve to illustrate the use of the tool.

The model building project generated component costs and supplier locations, and used these data to evaluate the choice of supplier using the GIS routing tool. The choice of supplier was evaluated in terms of cost and distance traveled for the construction material. These simple metrics demonstrate how the Multi-Origin Routing GIS tool can be used to support supply chain decisions. The heavy vehicle profile was used due to the transported materials being heavy and large objects. Using this profile can give more accurate routing. The distance metric can be further evaluated to calculate carbon emissions based on product weight and vehicle fuel consumption; however, this is beyond the scope of this study. The embodied carbon of the produced construction product can likewise be added into carbon impact calculations. For the purposes of demonstrating the utility of the tool, product cost and distance traveled are sufficient for showing how developers can have their decision-making supported by the GIS Multi-Origin Routing tool. Trade-offs between distance traveled and cost can be clearly visualized, and decisions can be supported by data to fulfill project needs.

The model-building project used an IFC building model of a large multi-level mixed-use building obtained from Ref. [52], which can be seen in Figure 5. IFC model files contain information on building elements, and IFC OpenShell [53] was used to generate an approximate material takeoff. The quantities of materials listed in the material takeoff were then used to estimate the sourcing cost of the materials obtained from suppliers at known locations using RSMeans [54]. RSMeans is a material sourcing cost estimation software used in the AEC industry.

The project is a multi-level mixed-use building. The building is seven storeys tall, and it has an approximately 365,000 sq ft surface area. Four material classes were selected, specifically cement, structural steel, rebar, and masonry, with five suppliers each. The multiple origin routing was computed for each class, which resulted in 4 multi-origin routing computations, or 20 total routing computations, respectively. This case study represents a typical envisioned use case for the Multi-Origin GIS Router. The case study was performed with a singular user use case on a standard desktop Windows 10 PC with an Intel Xeon 16-core processor. Routing computations were identical in perceived processing time to other mapping services such as Google Maps, MapQuest, and Bing Maps. A full measurement of response time is beyond the scope of this study; however, this represents an excellent avenue for future research.

The project site for the case study was selected to be at 100 Queen St W, Toronto. It is a central location in downtown Toronto in front of City Hall. The area is a prime location for large mixed-use construction projects, which can respond to multiple demands and needs of residents, including commercial shopping space and housing.

The quantities of materials obtained from the material takeoff were used alongside the addresses of known building material suppliers obtained from Ref. [51] to estimate material procurement costs. Building costs were obtained for construction materials of the modeled building in the city of Toronto region using the construction material price estimation software RSMeans [54]. These material costs were rescaled to the locations of the suppliers and their respective cities in order to obtain material costs from each individual supplier for each given material. Obtaining direct quotes would yield more accurate costs, but this would only be necessary for a project that is going ahead with construction, and it is beyond the scope of this exploratory case study. It should be noted that the costs are therefore estimations. This case study is primarily an instructive example of how the routing tool can be used to provide decision support, and how it can be incorporated into a BIM workflow.

The supplier origin addresses were taken from known construction material supply areas/industrial zones, which were also used in the case study presented in Ref. [51]. The quantities of required material can be seen in Table 2. The distance and the time for each supplier location to the proposed development site were calculated using the Multi-Origin GIS Routing Tool; however, only distance is reported in the interest of brevity. It should be noted that transportation costs are often based on the transportation distance, and the results of the routing can be further elaborated upon by calculating the transportation costs. This, however, requires in-depth real-world product supply data, which is beyond the scope of this study. Likewise, a complete BIM workflow would yield more accurate cost estimates for building materials. The current study is sufficient for demonstrating the use cases of the tool, and the method can be expanded upon by practitioners as needed. Additionally, the mixed-use multi-level building used in the case study required roughly 14 metric tons of concrete, among other materials, in addition to the four material classes selected for this case study. These additional materials have been omitted for the sake of brevity. Specifically, in the case of concrete, the suppliers were all located in close proximity within the same region, which made any difference in costs and distance traveled negligible.

The heavy vehicle profile in the Multi-Origin Routing tool was used for routing. This is due to the materials being transported via semi-truck and trailer transport. The dimensions of a standard semi-truck have inherent limitations, which affect routing, as some roads have restrictions both legislative and physical. The dimensions of a standard semi transport truck and trailer were obtained from Ref. [55] and entered into the profile such that the routing can account for the size of the transports. Additionally, the axel load was calculated using Equivalent Single Axel Load (ESAL) for a standard semi from Ref. [56]. For an axle load, 2.0 ESAL was selected for simplicity. The weight of 1.0 ESAL is 8.165 metric tonnes. Therefore, 2.0 ESAL = 16.33 Metric tonnes. The truck dimensions used in the study are summarized in Table 3.

### 4.1. Cement (Manufacturing/Terminal)

Supplier locations of five different suppliers were selected for cement. The cost breakdowns were estimated as described above, and the distances to the proposed development site were found using the Multi-Origin GIS Routing Tool. The cost vs. distance results are presented in Table 4.

The map in Figure 6 shows the geographic distribution of the suppliers to the construction site. The chart below in Figure 7 shows the cost vs. distance results for all the supplier locations. All of the suppliers are located along the Windsor–Quebec corridor. Supplier B presents the lowest cost option and the lowest distance traveled.

### 4.2. Structural Steel (Major Producers/Mills)

The impact of structural steel transportation and structural steel costs was compared from five known steel suppliers. The costs and distances are gathered in Table 5. The geographic spread of the suppliers can be seen in Figure 8. The distribution is varied, with some suppliers within the Windsor–Quebec corridor, some just outside it, and one located in northern Ontario.

The chart in Figure 9 compares the travel distances vs. suppliers. Suppliers A and B are virtually indistinguishable from each other relative to the scale of the other suppliers’ costs and distances traveled, with Supplier A being the slightly closer option. These two suppliers are the lowest travel distance options; however, these options also have the highest cost. Supplier E represents the best-balanced option between cost and distance traveled. Its travel distance is only 30 km higher than the nearest suppliers. Supplier D is the cheapest option but has a travel distance that is an order of magnitude greater than suppliers A and B. For a savings of roughly CAD 20,000, which equates to roughly a 1.3% difference from the highest cost option, the low cost of option D is not justified by the significantly greater travel distance. As such, unless budgets are especially limited, option E represents the best choice.

### 4.3. Rebar/Long Products (Mills + Fabrication)

An analysis was performed of the rebar suppliers in the case study to select the best option. All five known suppliers were compared against one another for trade-offs in terms of travel distance and cost, and the results can be seen in Table 6. A map of the supplier locations is presented in Figure 10.

All of the suppliers have costs between two and three thousand dollars. Their locations, however, are much more widespread. By examining the distance vs. cost chart in Figure 11 for rebar suppliers, it becomes evident that while distributor A is the lowest cost option, distributor C has only a negligibly higher cost and is located much nearer to the construction site than supplier A. As such, supplier C is the lowest distance traveled and the best balanced option between cost and distance traveled.

### 4.4. Masonry (Brick/Stone/Concrete Masonry Products)

Masonry suppliers were evaluated to determine which options had which benefits for the proposed project. The costs and travel distances of each supplier can be seen in Table 7. A geographic distribution of the masonry suppliers can be seen in the map in Figure 12.

Masonry presents some further options for sourcing due to there being different standards of bricks that are available for building within the construction industry. For this analysis, standard bricks of type 42210100400 and of type 42210100100 were selected, and these bricks have dimensions of 75 mm × 200 mm × 400 mm and of 50 mm × 200 mm × 400 mm, respectively. Costs were calculated for filling masonry requirements with both types of brick, and these costs are presented in Table 7 with the brick types of type 42210100400 and of type 42210100100, being contracted to #400 and #100, respectively. Between the two options, the #400 brick options were selected for a cost vs. distance traveled evaluation due to the brick being a lower price. The chart in Figure 13 shows the comparison of distance traveled vs. cost for each supplier using the #400 brick.

Distributors B, C, and D are all located within the same region and are priced the same using the RSMeans software [54]. These three suppliers are all within the area, balancing both lowest cost and lowest distance traveled in the distance vs. cost analysis. Suppliers B, C, and D all have less travel distance and cost than supplier E, and all three are significantly cheaper than supplier A while having a travel distance of between roughly 50km and 150km greater than supplier A. Within the three suppliers of B, C, and D, and within all suppliers, supplier B represents the best balanced option between cost and distance traveled, with the lowest cost and a travel distance that is only roughly 43 km greater than the minimum travel distance.

The results of the case study are summarized in Table 8. The choice of suppliers for each material is presented in terms of lowest costs, closest distances, and best balanced options. It can be seen that for some materials, the best balanced options, which balance cost and distance traveled, are also the lowest cost and/or the lowest distance options. This information is useful for decision makers and delivers the results of analyzing different options for suppliers at a glance, enabling quick review.

The performance of the tool was tested for responsiveness using the material routing for all four classes of materials. Performance was tested by calling the *echo %time%* function in the command line before and after routing. Timing was performed manually and is thus limited to human reaction time. As such, the values are somewhat slower than the true values. The results of the performance testing are presented in Table 9. Tests were conducted on 22 September 2026, EST.

All of the results are in the scale of seconds. This is excellent for usability and proves the responsiveness of the tool under typical small-scale user scenarios. Further performance testing would be needed to validate the performance of the tool under large-scale workloads. This is an excellent avenue for further study that would demonstrate the practicality of the tool at scale; however, it is beyond the scope and feasibility of the current study.

### 4.5. Further Application and Extension

This data can be analyzed and processed further by the user/practitioner, and it can be augmented to include carbon emissions by product creation, cost of transportation, product weight, and fuel consumption, among other parameters required to complete the carbon emission vs. cost analysis. This tool provides an efficient and powerful way to compare suppliers based on cost and distance traveled. This is an important first step, which can be expanded upon to compare environmental impact vs. costs with the additional relevant data. Its ease of use, the crisp presentation, and the usefulness of its results will be valuable in solving transportation logistics challenges and supporting decisions with data and measurable results.

The use of the tool can be further extended to use IoT sensor information from supplier vehicles. This would allow updating delivery times and rerouting delivery routes if information such as road hazards, heavy traffic, or road closures becomes known in the course of material delivery. IoT sensors can be utilized further to track the consumption of construction materials and the progress of their installation after delivery. This information can be relayed back to both the user and the BIM model and be leveraged for further supply selection decisions, progress monitoring, and schedule estimation.

### 4.6. Comparison to Existing GIS Tools and Multi-Origin Routing Methods

Current commercial software that provides routing falls into two broad categories: complete routing service systems for commercial shipping or delivery logistics, or GIS analysis software suites. Software in the first category is often geared towards fleet management services and is focused on multi-stop routing for transportation fleets [57,58,59,60,61], though there is no reason why this software could not also provide multi-origin routing. This software is highly specialized and requires subscription services in order to use. The second category refers to software suites used for GIS analytics primarily in research and academia, such as ArcGIS [62] and QGIS [63], with the former being a commercial software that is an industry standard, and the latter being a community-driven open-source alternative. These software suites provide multi-origin routing capabilities and, as in the case of QGIS, can link directly with the OpenRouteService library to perform routing, in the same way as was performed in the Multi-Origin GIS Routing tool. Furthermore, it is conceivable that, because it is free to use, research institutions, municipalities, and government agencies may use QGIS with OpenRouteService for multi-origin routing. As such, QGIS with OpenRouteService was selected for comparison against the Multi-Origin Routing tool presented in this work in order to show the advantages of our tool for logistics and decision support.

The first difference between QGIS and the Multi-Origin Routing tool is that QGIS is a software suite with a much larger install footprint, with many options and settings to configure, whereas the Multi-Origin Routing tool is a web app with a UI similar to Google Maps. Next, for routing in QGIS, the user needs to define layers with the address locations, which requires using a plugin alongside prepared lists of the addresses to geocode them onto a layer. It must also be noted that the geocoding in QGIS is performed with a plugin called MMQGIS, which uses OpenRouteService in the process, but the process is different than the geocoding performed directly with OpenRouteService in our Multi-Origin GIS Routing tool. The MMQGIS geocoding failed for certain addresses and required manual correction, which represents a further complication for a prospective user. In our Multi-Origin Routing tool, geocoding is performed automatically, and the user just enters the addresses in question. In QGIS, the user has to use the OpenRouteService plugin to route between the origins in a specified origins layer and the destination in a specified destinations layer, and must select the all-to-all option to ensure that each origin is paired with the destination. Once again, this is much more complicated than the process in the Multi-Origin Routing tool, where this is all performed in the backend once the user requests a route to be calculated. The routing that was performed with the Multi-Origin GIS Routing tool was repeated with QGIS and the OpenRouteService plugin. Special care was taken to select the same parameters as much as possible, such as the heavy vehicle driving profile. The QGIS interface did not allow for truck dimensions to be specified; however, this represents a limitation for the QGIS approach. The results of the routing for each material class were compared and are presented side by side in Figure 14.

The results are almost identical in almost all cases, with the exception of the structural steel material routing, which changed the route to go through Michigan in the US rather than through Sudbury. These generally minor variations, despite using the same GIS routing library, can be explained by the fact that settings were set in marginally different ways in the backend of both software. In addition, the routing layers need to be saved to be made permanent once they are generated in QGIS, and statistics on route distances must be obtained by opening the layer attribute table. This is significantly more complex than the results generated in the Multi-Origin routing tool, where results are automatically presented in a clear and concise table within the UI. All of this demonstrates that QGIS is fully capable of multi-origin routing for logistics support. However, the degree of difficulty is much larger, and users require special training and field expertise to navigate all of the analysis options and to obtain consistent results.

Broadly comparing the Multi-Origin GIS routing tool to other GIS tools used for routing by other research studies reveals some further benefits of the tool. A comparison of features of the Multi-Origin GIS routing tool to other web-app mapping and GIS tools can be seen in Table 10. From examination of the table, it can be seen that only the Multi-Origin GIS router tool has a simple-to-use web-app interface and provides multi-origin routing for logistics without requiring data preparation and domain knowledge of how to use GIS analytics software. Among the different types of tools used, most studies use research-oriented GIS analysis software for their applications due to the analysis requirements of their studies. It should be noted that many studies do not explicitly mention the specific tools used for routing and spatial analysis. However, if the study requires sufficiently complex geographical data, it is a safe assumption that the work used either ArcGIS or QGIS or some analogous GIS software. The key drawback to this software, and the key argument for the creation of the Multi-Origin GIS Routing tool, is that this software is difficult to use for users without domain knowledge, has a difficult-to-use user interface, and additionally requires layer data, all of which is beyond the scope and abilities of the regular user in low-digitalization environments where GIS analytics are acutely needed to make informed project and policy decisions.

The Multi-Origin GIS Routing tool is a much simpler-to-use web application with a UI similar to Google Maps. The tool distills arguably the most important part of GIS analytics for logistics into a package that a non-expert can use to quickly obtain multi-origin routing analysis, and a practitioner could incorporate easily into a BIM workflow for decision support and visualization. These differences are primarily due to the structure of general-purpose GIS software suites versus the application-specific GIS web app tool. The Multi-Origin Router lacks features that are found in QGIS and other GIS analytics software suites that enable a wide variety of analyses, but that is the main reason for its usefulness, which is ease of use and focus on, and quick setup for, logistics routing analysis.

## 5. Discussion

The case study shows the analytical advantages that can be brought to logistics projects by the Multi-Origin Routing tool. Visualizing transportation routes, seeing the distance traveled alongside the costs, being able to assess the scale of trade-offs, and supporting decisions with data are significant project benefits. These benefits are realized by using the tool and by processing the produced results. Multi-origin routing has applications in a range of aspects related to built asset projects. Multi-origin routing can be used for decision support for construction and rehabilitation phases in a building life cycle. It can also have conceivable applications for demolition and disposal if the destination is treated as the origin, and the origins are treated as destinations in the impact vs. costs analysis.

The Multi-Origin Routing tool is a first step in achieving BIM-GIS integration. Bidirectional synchronization between BIM models and GIS data, IoT integration, and digital twin functionality are further avenues of development that arise from this tool and the presented workflow. This is an initial level of BIM-GIS integration through BIM-assisted GIS routing. It serves as a starting point for organizations interested in leveraging the benefits of GIS, and will require further development to achieve complete digital twin implementation.

The Multi-Origin Routing tool is built using existing open-source technologies. The packaging of these technologies to allow users to use multi-origin routing GIS analytics in an easy-to-use web-app interface seems to be an obvious step toward democratizing GIS analytics in the public asset management and AEC fields. However, this packaging has not yet been performed, or if it has, it is not readily available for users. The innovation of the Multi-Origin Routing tool is that it packages highly useful multi-origin routing analysis into a web-app mapping application which users without software development or GIS domain knowledge can use as they would Google Maps, MapQuest, or Bing Maps. The packaging of these mechanisms is the chief labor behind this project. Though this may appear to be an easy next step in the development of existing open-source resources, the details of working with web-app interfaces and GIS mapping servers are beyond the scope of most users and many organizations that deal with public asset management and AEC projects for government authorities, as well as other AEC projects in the low-digitization construction industry. As such, this tool will be a welcome addition to supply chain and logistics management.

The decision support provided by the tool, and by analyzing the results, allows AEC practitioners and other logistics practitioners to weigh the benefits and drawbacks between different decisions. Evaluating the costs, distance traveled, and calculating the emissions of different solutions is valuable for decision makers to be able to balance different project needs and to choose efficient logistics options.

As was evident in the case study, different options often come with different trade-offs. This follows because there are two objectives in this particular case study, and a supplier choice may be better in one objective than another compared to the competing options. In multi-objective optimization, the set of options that are better for all objectives relative to the weight of one vs. the other is located along the lower left of the objectives graph, where the lowest values for both objectives are found. This is known as a Pareto front. These options beat all other options such that, for a given option, no other option is better than it in terms of every objective. The multi-objective routing tool allows practitioners to find the Pareto fronts of logistics options. These fronts represent the most efficient solutions relative to all the objectives and allow for an informed decision to be made relative to which objective is more important and how much weight to assign to the second objective. Inefficient solutions, which are ineffective in terms of costs and reducing environmental impact via travel distance, can be discarded, and efficient solutions can be selected in their place.

The path routing provides a solid basis for environmental impact analysis. It can be further combined with carbon emissions per traveled kilometer due to fuel consumption and weight, and with carbon associated with manufacturing and transportation costs, for a more complete impact vs. cost analysis.

The tool is an easy-to-use web app that provides a GIS interface for multi-origin routing. While similar effects can be achieved with costly or complex software solutions, the easy-to-use web application is free for use within reasonable limits, and it requires virtually no software development knowledge. As such, the range of users that can use this tool to examine and solve logistics problems is much larger.

The tool can be used for simulation and routing in a simulation-optimization framework. The optimization methods can call the tool via the programmable API to optimize a supply chain based on different available material sources. As such, the GIS-powered analytics can be used in optimizations to evaluate proposed solutions and converge toward supply chain improvements.

There are significant advantages that this tool has over similar tools and workflows. It is not only easy to use, but also easy to install and portable. In the low-digitization environment of AEC, this makes use and adoption dramatically easier. Multi-origin routing allows for basic GIS analytics to be added to new or existing projects. This allows users to begin to leverage some of the benefits of GIS immediately. It is a simple starting point that allows users and organizations to incorporate GIS for a wide variety of projects. This tool provides a base level of GIS analytics that can be improved upon for further applications, depending on user and organizational needs and means. The ease of use of the tool has thus far only been evaluated informally. Verifying it formally with user experience surveys comparing different tools and workflows is an excellent direction for further research. The formal assessment would yield valuable insights into improving usability in BIM-GIS workflows and will assess where the tool’s strengths and weaknesses lie for user experience. Thus, improvements can be directed towards user needs, which will improve adoption. Formal evaluation of usability would be an instructive part of capacity building within organizations that will, as a consequence, train new users to use the software for BIM-GIS-enabled logistics.

Within certain limits, the tool is free to use and open source. This allows the conservation of public budgets while leveraging the benefits of routing in GIS. Governments and government agencies, as well as AEC practitioners who have low levels of digitization or limited budgets in their organizations, can use this tool as a starting point. The tool can help to optimize their supply chains and leverage the benefits of digitization with their existing workforce and without added software costs.

This work has demonstrated how BIM-assisted GIS-based logistics decisions can be made using the tool. Further work is needed to achieve a full integration with BIM for complete two-way scheduling and prediction, such that changes in the BIM model reflect supplier and routing decisions, and conversely, changes in real-world transportation conditions such as traffic delays impact the predicted schedule of completion within the BIM process. This limitation can be addressed by routing using real-time sensor-driven data from IoT sensors and integrating it into BIM processes for accurate scheduling and enhanced decision support. Moving beyond the current level of BIM-assisted GIS routing would allow users to make supplier decisions based on evolving project needs, and scheduling can be updated to accurately reflect anticipated arrival times. These developments would improve decision support and analyses for further real-world case studies as well as show how the tool can be used for practical improvement in capital expenditure efficiency. Comparing the impact of decisions with and without updated situation information from BIM-GIS integration will be impactful for further research work. BIM-GIS-enabled tracking of delivery and installation of building materials will show how improvements in scheduling accuracy and material tracking will yield cost savings, and allow planners and operators to make trade-off decisions between costs and environmental impacts. As such, further development of BIM-GIS integration using the Multi-Origin GIS routing tool will yield incremental cost and impact assessment benefits, as more information channels become established between BIM models and real-world assets.

An important limitation of this tool is that it has only been validated on case studies of a single scale in one geographic area. Further validation of the tool is necessary to assess its practical use for construction projects of different scales, and how its routing is affected by differences in geography. Conducting additional case studies of differing size, location, and supplier requirements is an excellent direction for future research that will validate the usefulness of the tool and will allow scaling challenges to be addressed to improve the robustness of the system and draw more users to use it for logistics projects. The effect of different transportation schemes is particularly useful for further study, such that different shipping approaches and their impacts can be examined. Contacting suppliers for actual shipping quotes, while laborious, may also prove to be useful for differentiating suppliers within the same geographic area, and would lead to more accurate cost estimation. The labor involved in obtaining price quotes from suppliers may be reduced using Generative AI (GenAI), and this should be further explored to streamline efforts in logistics for BIM-GIS decision support. Demonstrating more accurate material cost impacts of decisions will serve to convince technologically skeptical industry practitioners of the value of the tool and is an important avenue of future study that will be amongst the most impactful. Research innovation only leaves research and moves to industry when economic incentives are validated.

This tool is limited by the accuracy of OpenRouteService’s maps. In areas where the mapping is less accurate, the distance traveled and the routing will be less reliable. In addition, cost uncertainty due to changing supplier conditions, as well as changes in the project requirements during construction and due to hazards and emergencies on transport routes, will all have impacts both on cost and on routing. Exploring the impacts of these challenges, measuring the outcomes on costs and routing, and finding mitigation measures for risks will demonstrate the robustness of the tool to real-world conditions and measure its practicality for logistics planning. How these challenges can be addressed using different mapping services and backup suppliers is a needed direction for further study to validate the tool. As such, performing real-world case studies in differing locations, with costing quotes, and specific transport characteristics, is the most important direction for future research.

The tool was tested in singular case studies with singular user use cases. Its performance was identical to that of systems such as Google Maps under similar use cases. However, it remains to be seen how performance scales with larger task loads, and a full examination of different sizes of workloads with different numbers of users is a necessary step for future investigation to determine the performance of the system under different user loads. Metrics such as route computation time, system response time, and data processing time should be evaluated under case studies of differing sizes, with individual and simultaneous users. It is important to understand the limits of the free use allowances for organizations looking to use this tool and scale it to large projects. Use beyond the 2000 daily routing requests or 10,000 daily requests for government agencies [50] requires on-premises hosting of OpenRouteService. This means that organizations looking to scale the tool must account for its success and plan to allocate resources for hosting servers for the required libraries and web services. The challenges of setting up such services and integrating them with BIM processes are transformative, and the implementation would be of interest to the research community and to AEC practitioners to demonstrate how the scalability would be achieved in practice.

The Multi-Origin GIS Router was compared to the competing free-to-use GIS software suite of QGIS with the OpenRouteService plugin. The results were almost identical for almost all routing cases, and the differences can be explained by internal use settings. The results provided by the Multi-Origin Router were just as useful as the ones that were produced with QGIS. The structure and the complexity of both software are where the key difference in performance lies. In the low digitization environment of AEC and municipal asset management, the need is for useful tools that everyone can use, which are geared to the needs of logistics and decision support. The needs of this user environment are not served by general-purpose and complex software suites that must be tuned with specialized software knowledge and experience in GIS. The cost–benefit analysis of the different suppliers can be performed with the results from either software. However, the Multi-Origin GIS Router provides results more easily and faster, with a better user interface pre-configured for logistical decision support.

The tool provides routing analytics for construction supply chains in the form of a free, ready-to-use software that is highly portable and able to be integrated into further applications via a programmable API. The highly portable web-app format means that it can run on most computers without any specialized software. Other routing tools exist, but they have additional limitations, not the least of which is cost, that our routing tool does not have. A UI for multi-origin GIS-based routing, similar to Google Maps, does not, to the best of our knowledge, exist elsewhere; however, it is needed within AEC and public asset management.

## 6. Conclusions and Future Work

A Multi-Origin GIS Routing Web Tool is presented for solving logistics and supply chain problems and challenges. The tool provides users with analytics and visualization to weigh different supplier options for construction and rehabilitation projects on built assets.

The Multi-Origin GIS Routing Web Tool represents an initial step toward BIM-GIS integration, with BIM-assisted GIS routing. Further development will be needed to achieve the full capabilities of BIM-GIS integration, including bidirectional synchronization between BIM and GIS, IoT integration, and digital twin functionality. This initial level of integration is a good starting point for organizations looking to implement BIM-GIS workflows because it makes commencement feasible and allows for immediate use of GIS technology for routing.

A case study is presented to show a practical example of how the tool can be used. The visualization and the produced results allow users to weigh distances vs. product costs, and can be further analyzed to evaluate environmental impact vs. combined product and transportation costs. The tool comes in a simple-to-install and use web application that serves as an easy entry point for GIS analytics for a wide range of users, including those with limited experience. The tool is free to use within reasonable limits, and it is open source, allowing for budget-constrained public organizations to conserve funds and to be market-impartial. Comparisons to GIS software suites demonstrate that it is an improvement in user interface and usability, especially for non-experts.

Future research directions include validating the Multi-Origin Routing tool using more construction projects, obtaining price quotes from individual suppliers for more accurate cost assessment, perhaps using GenAI automation for email contact, and incorporating environmental impact assessment of different supplier and material choices. Further development of this tool requires performance testing on more case studies in varying geographical areas and different user workloads. A deeper integration of BIM for two-way information exchange on scheduling and decision support between routed supplies and the BIM model needs to be studied to leverage the full benefits of BIM-GIS integration. This tool offers an initial step toward BIM-GIS integration with BIM-assisted GIS routing and, as such, offers a platform that facilitates further research towards leveraging the benefits of cost assessment, impact assessment, and progress tracking using BIM models and IoT sensor tracking of physical assets.

Additional research could explore adding affected area analysis to bring more GIS capabilities to low-complexity web-based mapping tools. There are also applications for inverting the routing from multi-origin to multi-destination for use with other logistical routing challenges such as waste disposal and multi-site material delivery for large-scale projects. Further GIS analytics methods could also be added by building upon the extended GIS functionality of the OpenRouteService library. In addition, the adoption of this tool could be encouraged by establishing community maintenance and development.

There are plans to incorporate the Multi-Origin Routing tool into BIM-GIS integration projects for the National Research Council of Canada and our research partners. As an initial form of BIM–GIS integration, the tool associates the geographic location and project context of a BIM model with GIS-based analysis of material suppliers, transportation routes, travel distances, and estimated travel times. This enables BIM-informed logistics options to be evaluated and visualized geographically. This tool can be used to create decision support systems, allowing decisions involved in costs and impact trade-off considerations to be supported by data, which allows for clear communication and understanding. These decision support systems can improve the productivity and sustainability of building, maintenance, and rehabilitation projects. As governments and government agencies tend to have substantial portfolios of built assets to manage, there will be significant applicability and interest in leveraging the benefits of digitization, BIM, and GIS. The Multi-Origin GIS Routing tool makes it possible to do so in a way that is intuitive, efficient, and cost-effective.

Users and practitioners who are interested in using the tool are encouraged to contact the authors at the coordinates provided.

## Figures and Tables

**Figure 1:**
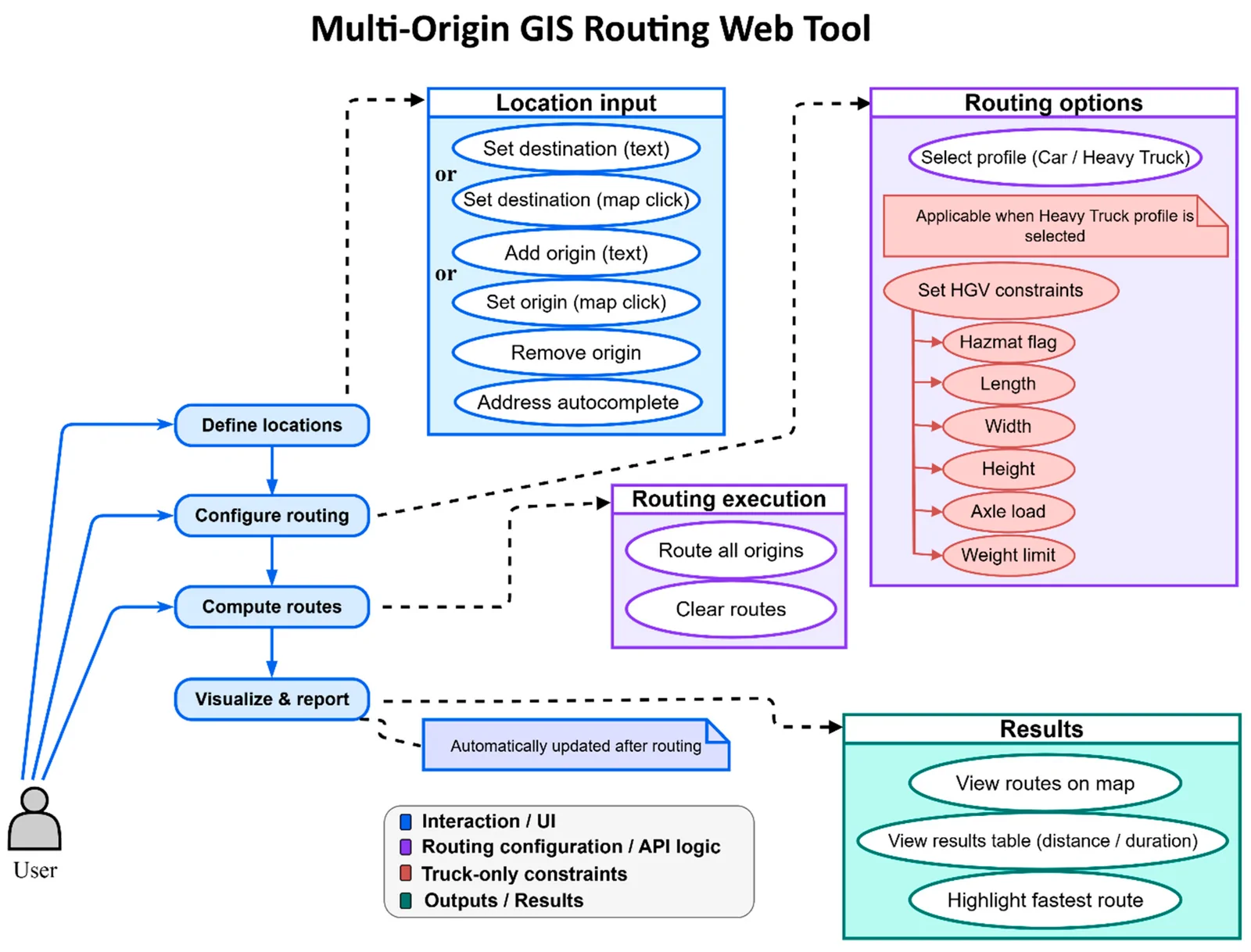
Multi-Origin GIS Routing Webtool deployment diagram.

**Figure 2:**
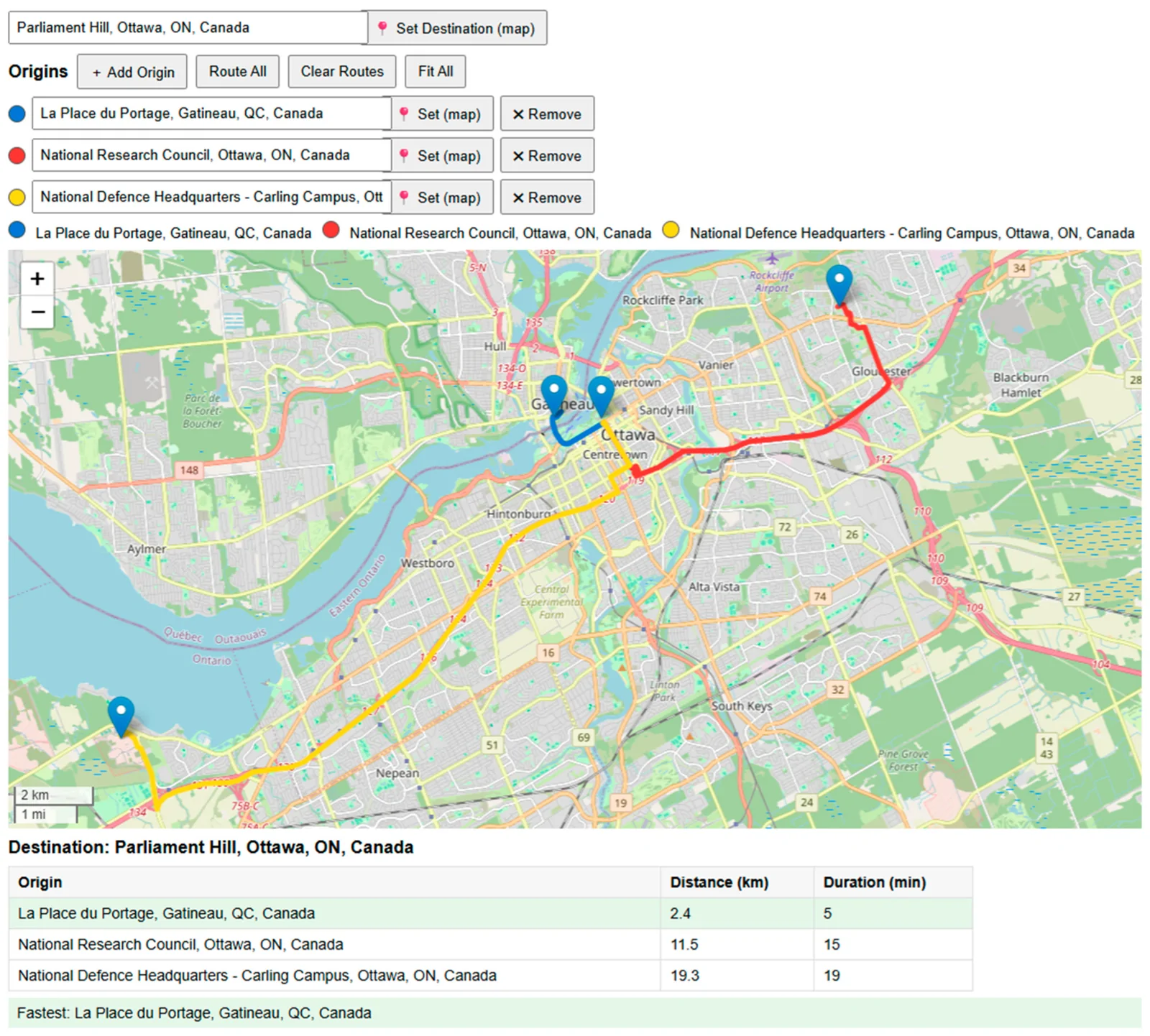
Web-based user interface of the Multi-Origin GIS Routing Tool.

**Figure 3:**
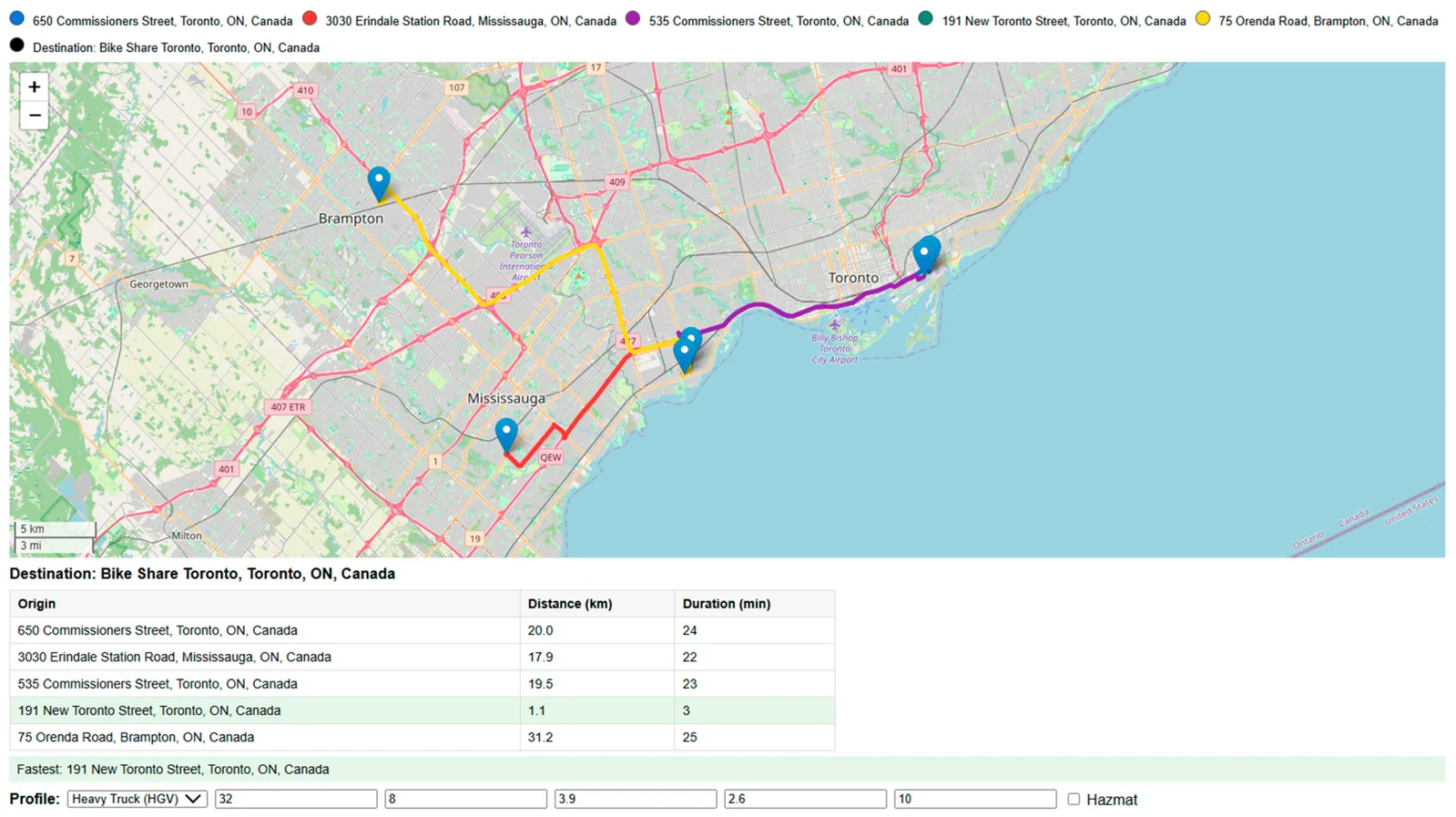
HGV profile inputs, including optional vehicle constraints and HAZMAT flag.

**Figure 4:**
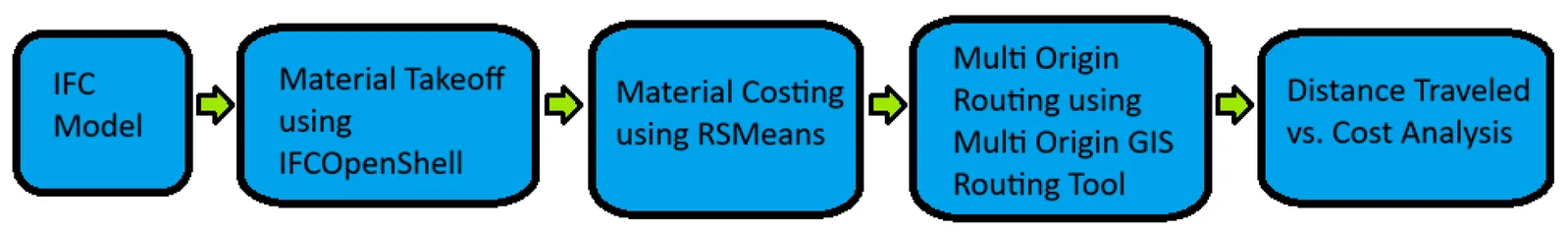
Material supplier selection using Multi-Origin GIS Routing in a simulated BIM workflow.

**Figure 5:**
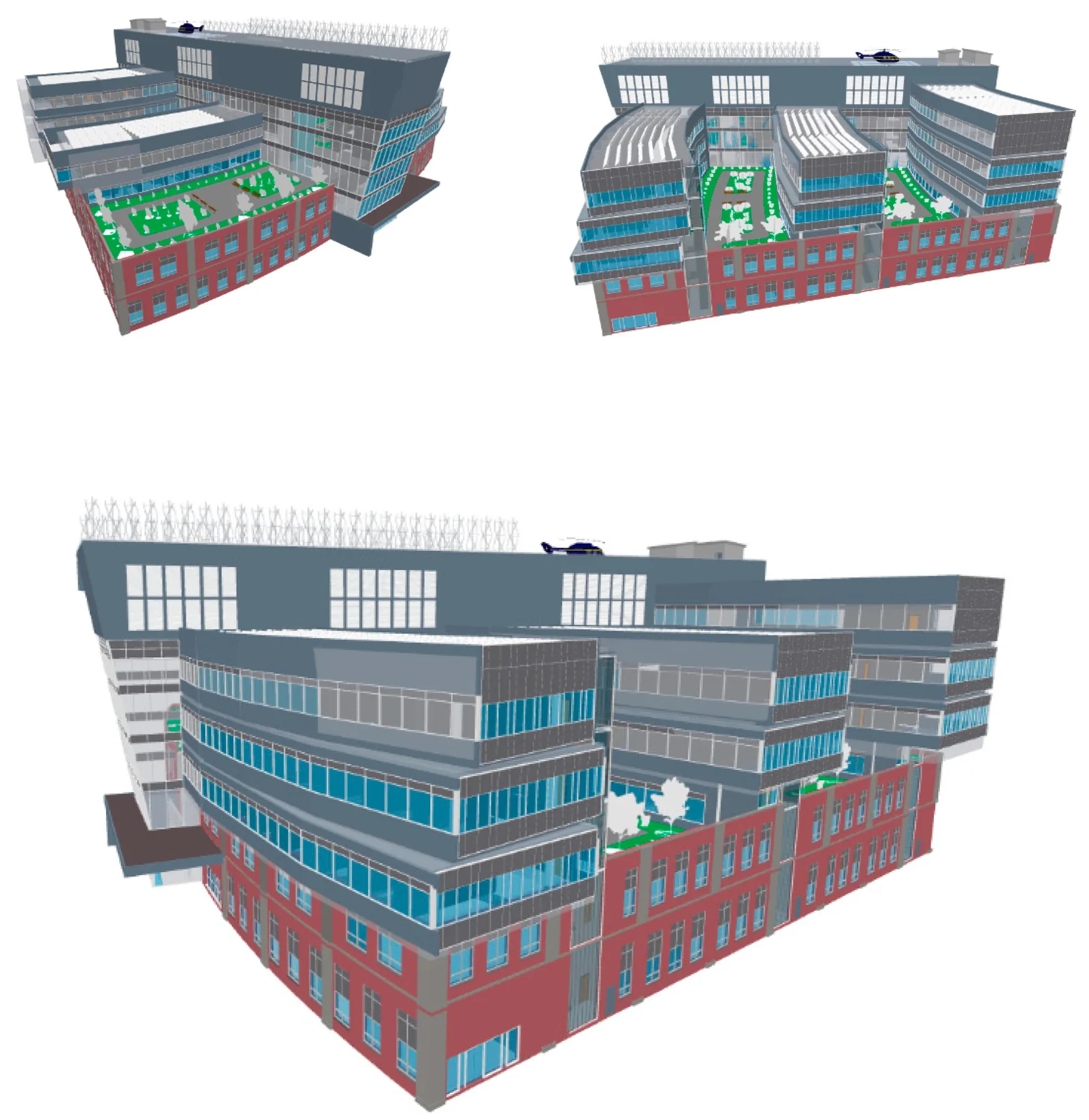
Mixed-use multi-level building used for case study. Views obtained from IFC building model [52].

**Figure 6:**
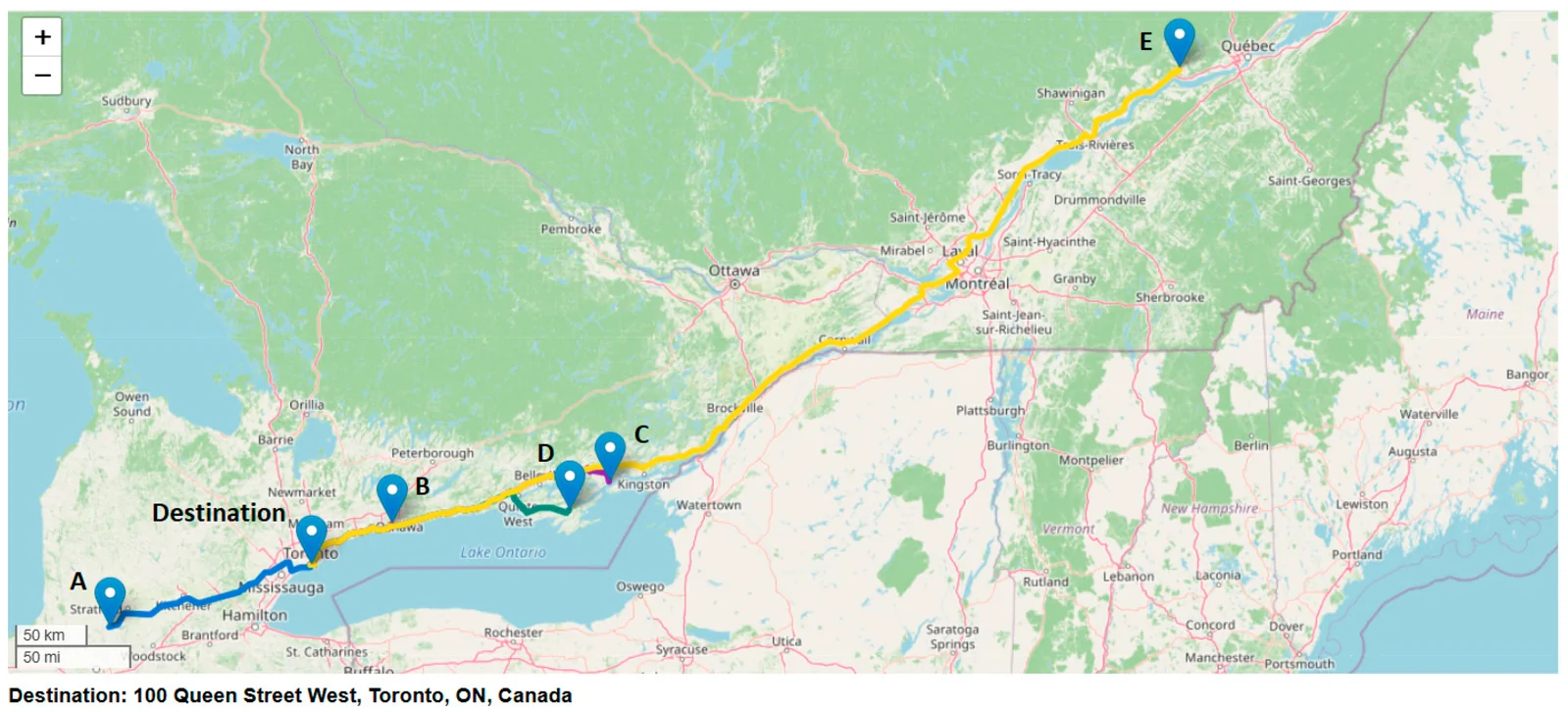
Cement suppliers map. Routes to suppliers A, B, C, D, and E are indicated by blue, red, purple, green, and yellow, respectively. Distances are indicated in Table 4.

**Figure 7:**
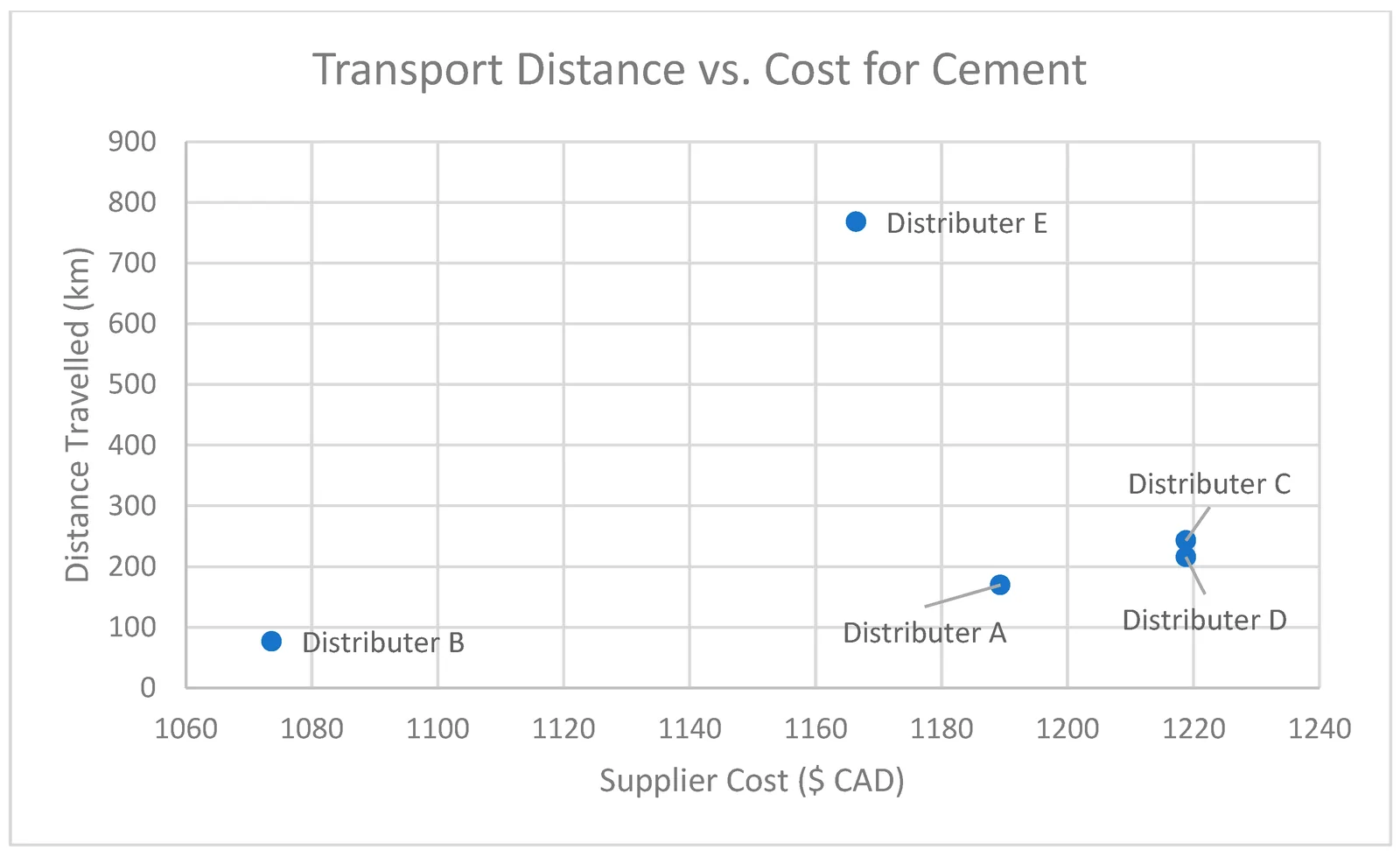
Transport distances vs. cost chart for cement suppliers.

**Figure 8:**
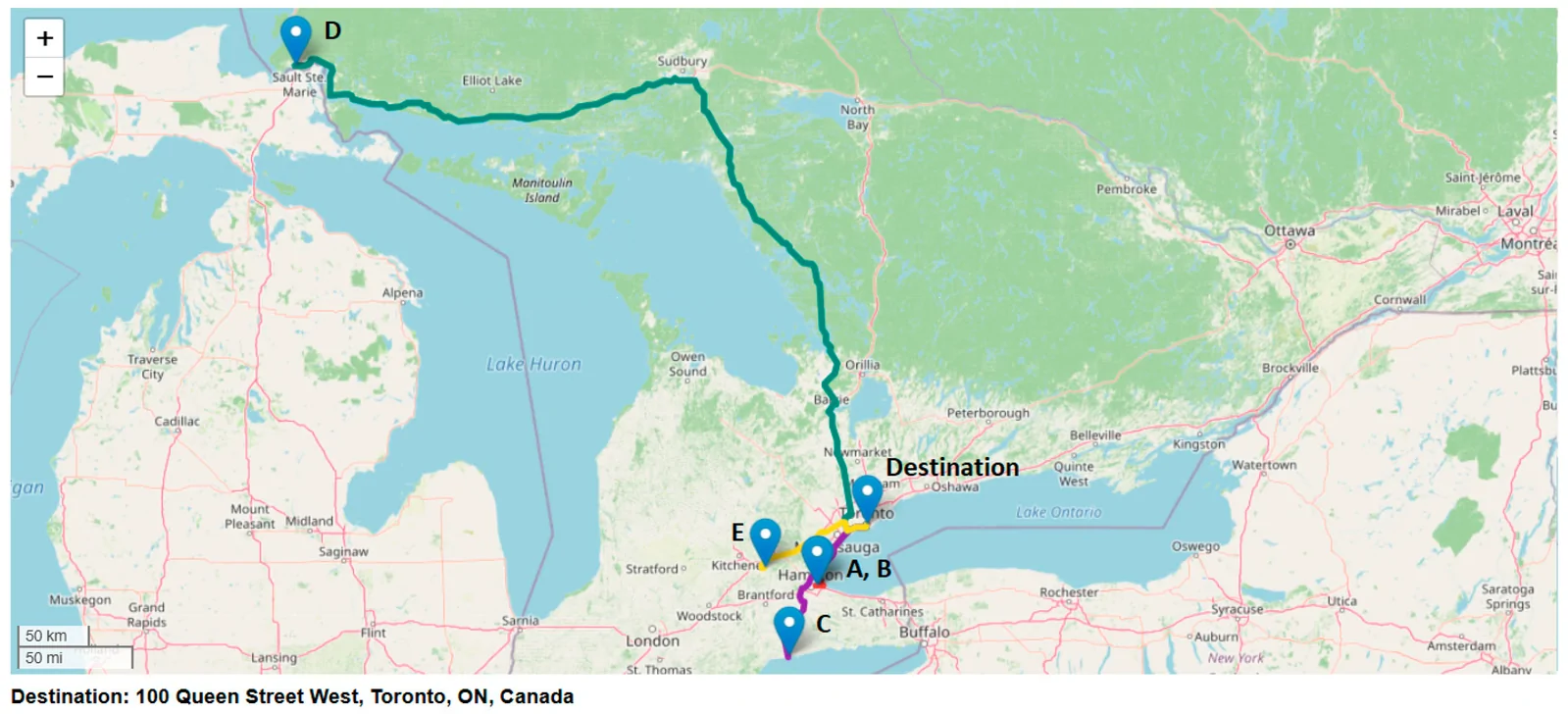
Structural steel suppliers map. Routes to suppliers A, B, C, D, and E are indicated by blue, red, purple, green, and yellow, respectively. Distances are indicated in Table 5.

**Figure 9:**
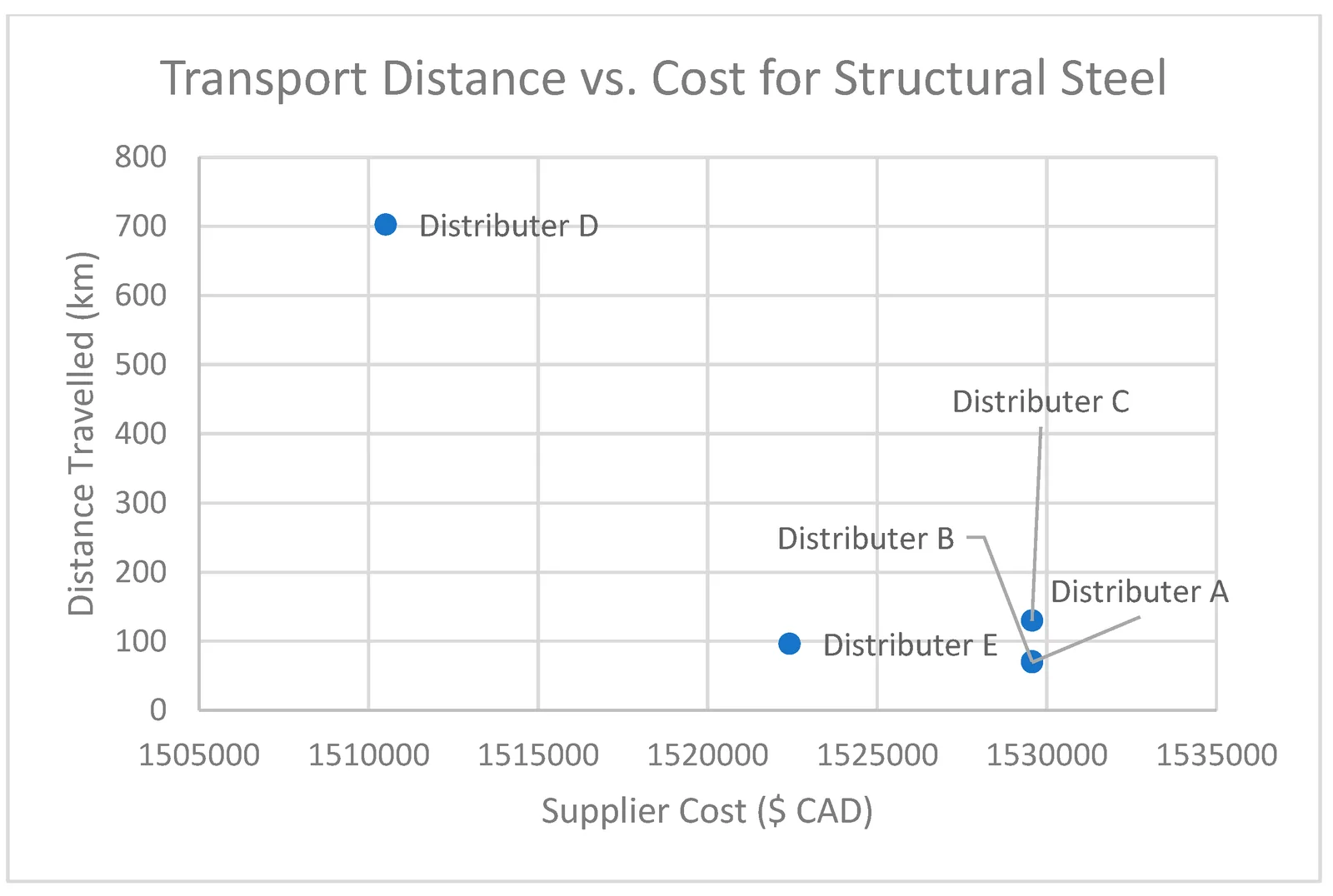
Transport distances vs. cost chart for structural steel suppliers.

**Figure 10:**
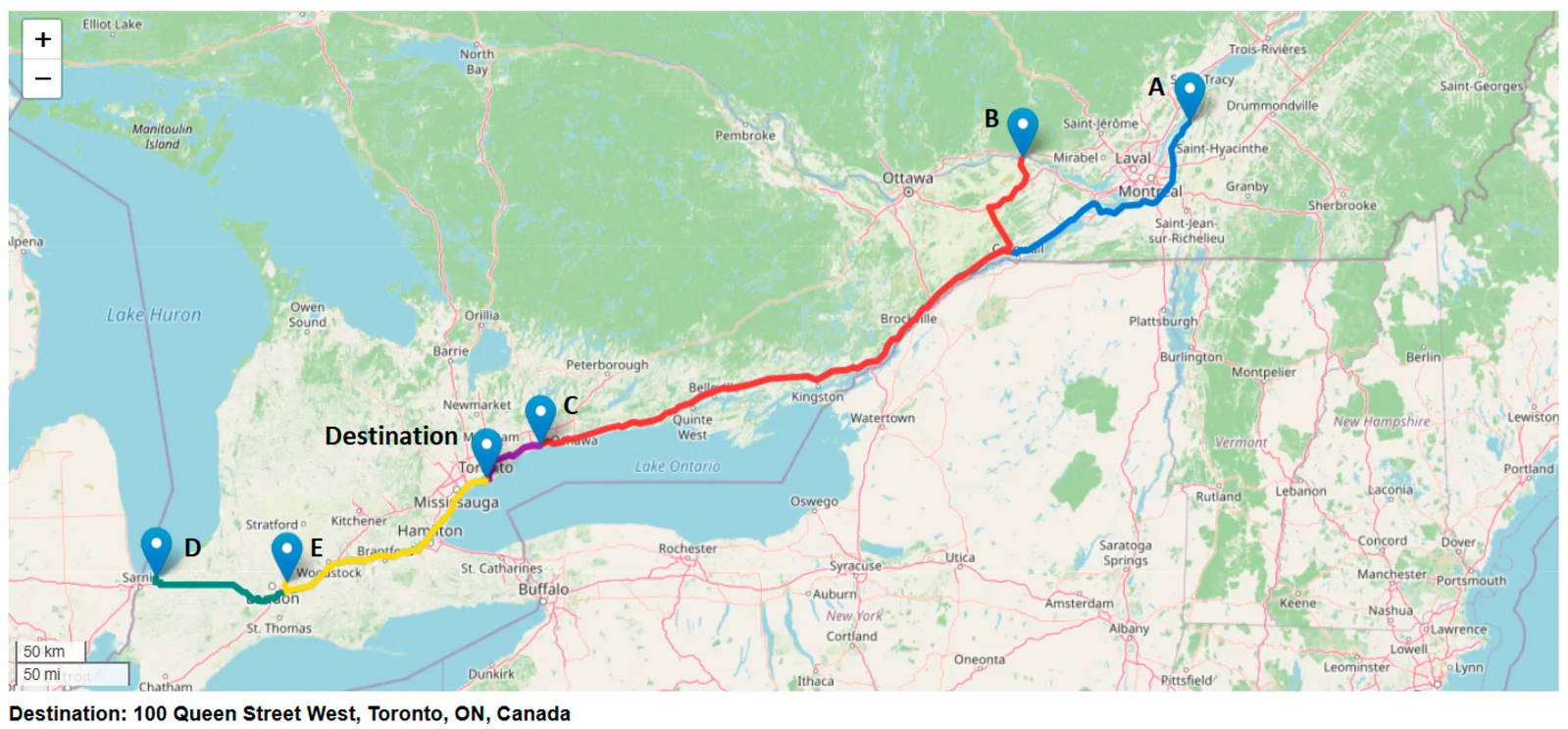
Rebar suppliers map. Routes to suppliers A, B, C, D, and E are indicated by blue, red, purple, green, and yellow, respectively. Distances are indicated in Table 6.

**Figure 11:**
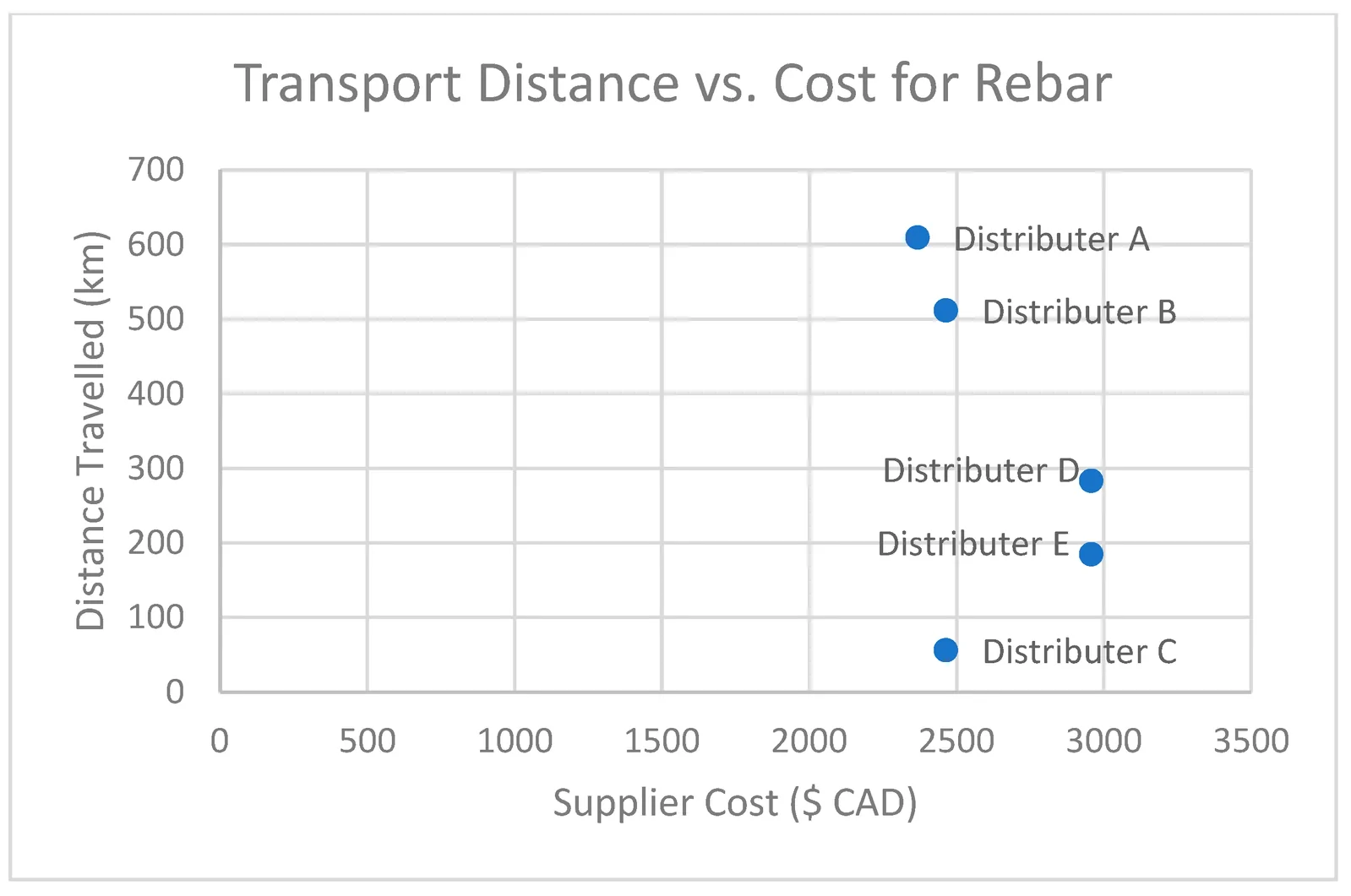
Transport distances vs. cost chart for rebar suppliers.

**Figure 12:**
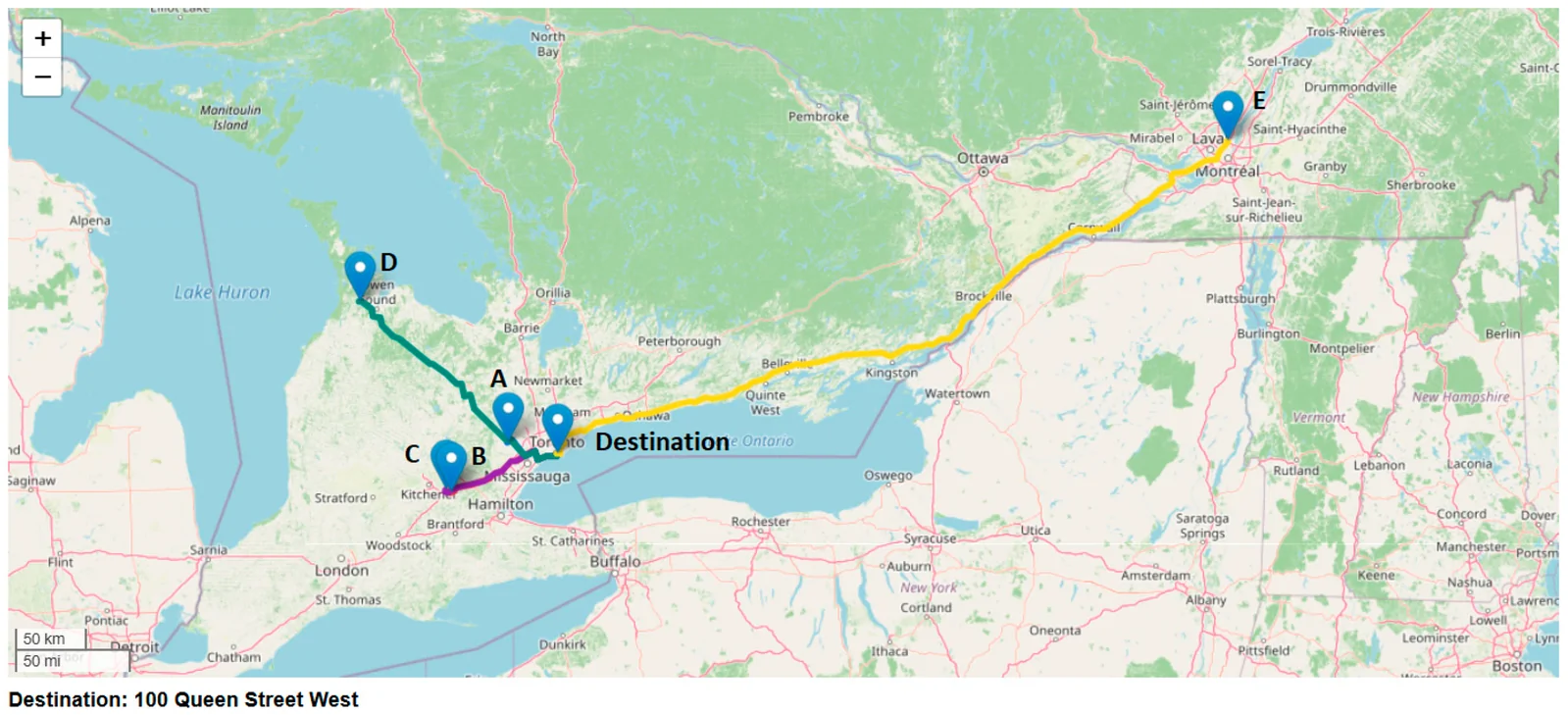
Masonry suppliers map. Routes to suppliers A, B, C, D, and E are indicated by blue, red, purple, green, and yellow, respectively. Distances are indicated in Table 7.

**Figure 13:**
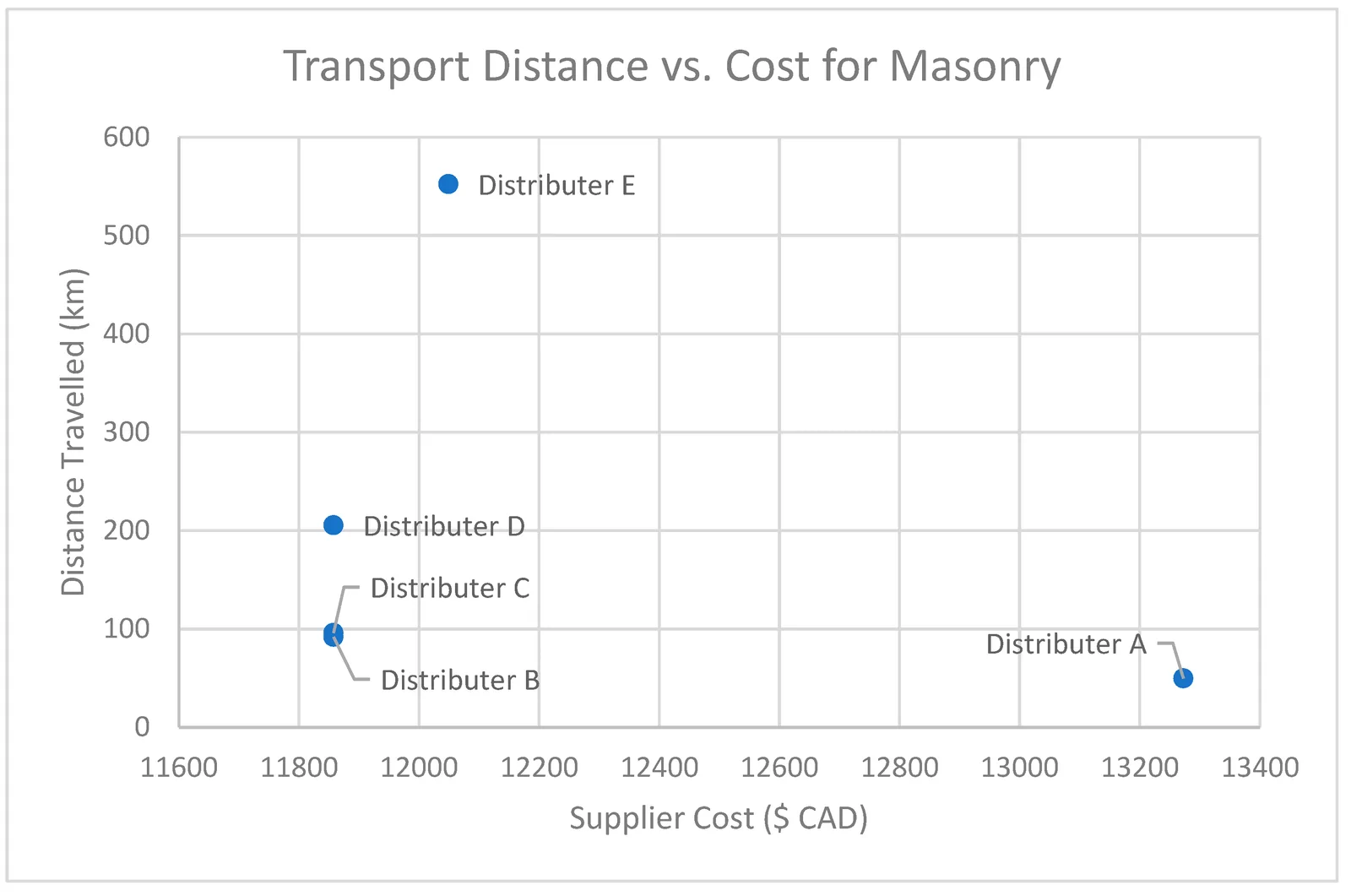
Transport distances vs. cost chart for masonry suppliers.

**Figure 14:**
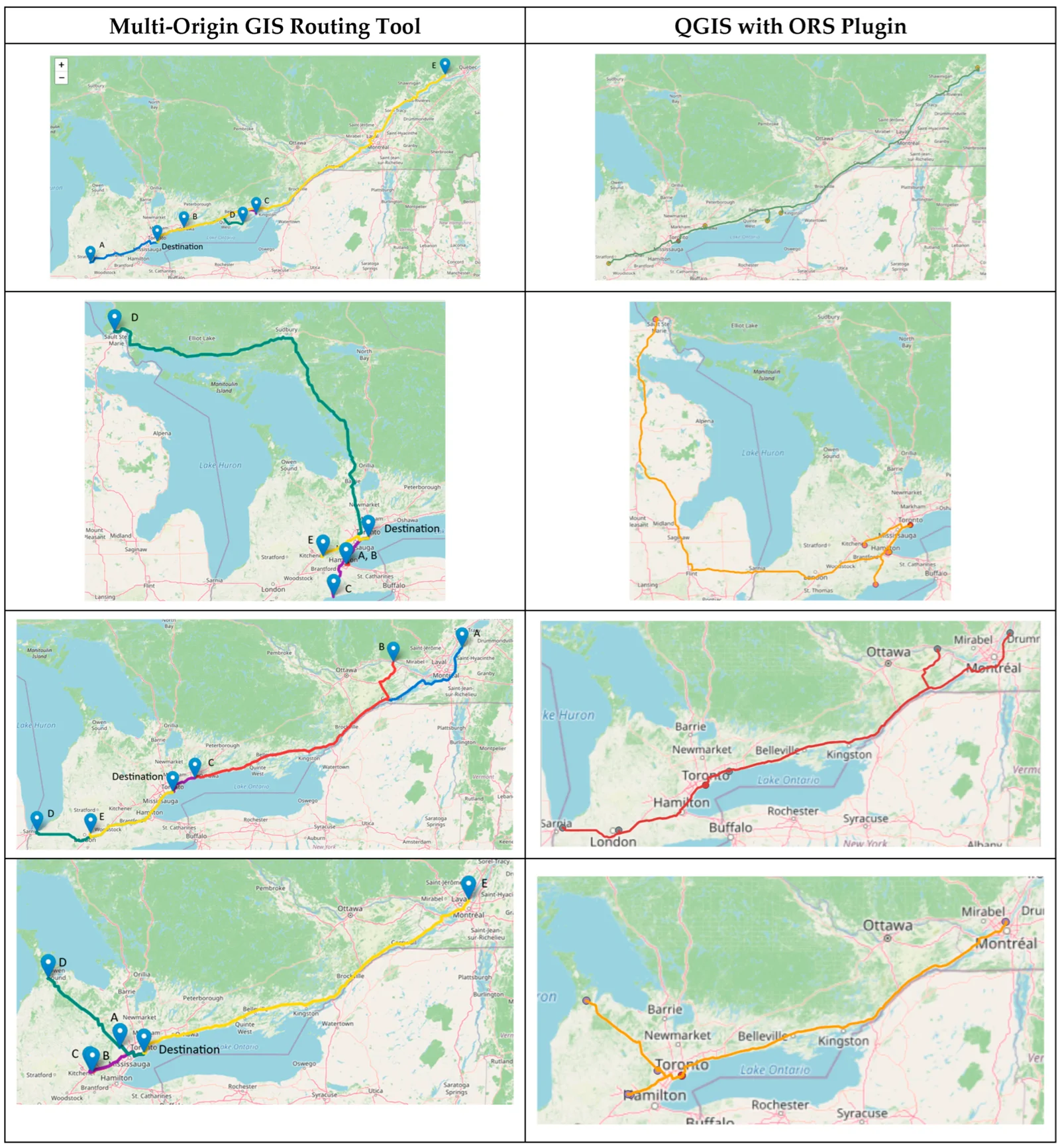
Side-by-side comparison of Multi-Origin GIS Routing Tool and QGIS with ORS Plugin for multi-supplier logistics routing. **First Row** = Cement Suppliers; **Second Row** = Structural Steel Suppliers; **Third Row** = Rebar Suppliers; **Fourth Row** = Masonry Suppliers. For left column maps from the Multi-Origin Routing tool, routes to suppliers A, B, C, D, and E are indicated by blue, red, purple, green, and yellow, respectively, same as the case study maps. For the right column maps from QGIS, the routes to suppliers are indicated in coloured paths that all share the same colour. Distances are omitted. Only route structural comparison was needed for the comparison. For information on distances, and supplier locations, consult the case study earlier in this section.

**Table 2:** Material quantities required for building takeoff.

Material	Estimated Mass (t)
Cement	1.892
Structural steel	238.25
Rebar	0.58
Masonry	43.6

**Table 3:** Heavy vehicle profile dimensions.

Dimension	Quantity
Height (m)	4.12 m
Width (m)	2.6 m
Length (m)	22 m
Axel Load (t)	2.0 ESAL = 16.33 t

**Table 4:** Location vs. *Distance* and *Cost* for cement suppliers.

Distributer	Address	Distance (km)	Cost ($ CAD)
Distributer A	585 Water Street South, St. Marys, ON, N4X 1B6	169.6	1189.32
Distributer B	410 Bowmanville Avenue, Bowmanville, ON, L1C 7B5	76.7	1073.6
Distributer C	6501 Bath Rd, Bath, ON, K0H 1G0	243.1	1218.8
Distributer D	1370 Highway 49, Picton, ON, K0K 2T0	215.6	1218.8
Distributer E	145 boulevard du Centenaire, Saint-Basile, QC, G0A 3G0	768.4	1166.44

**Table 5:** Location vs. *Distance* and *Cost* for structural steel suppliers.

Distributer	Address	Distance (km)	Cost ($ CAD)
Distributer A	1330 Burlington Street E, Hamilton, ON, L8N 3J5	69.7	1,529,565
Distributer B	386 Wilcox Street, Hamilton, ON, L8L 8K5	71	1,529,565
Distributer C	2330 Regional Road #3, Nanticoke, ON, N0A 1L0	129.8	1,529,565
Distributer D	105 West Street, Sault Ste. Marie, ON, P6A 7B4	702.6	1,510,505
Distributer E	160 Orion, Cambridge, ON, N1T 1R9	96.4	1,522,417.5

**Table 6:** Location vs. *Distance* and *Cost* for rebar suppliers.

Distributer	Address	Distance (km)	Cost (CAD)
Distributer A	4000 route des Aciéries, Contrecoeur, QC, J0L 1C0	609.5	2367.27
Distributer B	1040 Hwy 17, L’Orignal, ON, K0B 1K0	511.6	2463.84
Distributer C	1 Gerdau Court, Whitby, ON, L1N 5T1	56	2463.84
Distributer D	318 Arvin Ave, Stoney Creek, ON, L8E 2M2	283	2957.13
Distributer E	408 Neptune Crescent, London, ON, N6M 1A1	184.7	2957.13

**Table 7:** Location vs. *Distance* and *Cost* for masonry suppliers.

Distributer	Address	Distance (km)	Cost Using #400 Brick (CAD)	Cost Using #100 Brick (CAD)
Distributer A	225 Wanless Drive, Brampton, ON, L7A 1E9	49.8	13,272.75	18,246.84
Distributer B	200 Sheldon Drive, Cambridge, ON, N1T 1A5	92	11,857.5	16,238.54
Distributer C	875 Speedsville Road, Cambridge, ON	96	11,857.5	16,238.54
Distributer D	281,227 Shouldice Block Rd, Uxbridge, ON, L9P 1R4	205.5	11,857.5	16,238.54
Distributer E	8145 rue Bombardier, Anjour, QC, H1J 3G5	552.3	12,048.75	16,468.06

**Table 8:** Results of supplier selection for case study per material.

Material/Decision Option	Supplier	Distance (km)	Cost (CAD)
**Cement**			
Lowest Cost	Supplier B	76.7	1073.6
Closest	Supplier B	76.7	1073.6
Best Balanced Option	Supplier B	76.7	1073.6
**Structural Steel**			
Lowest Cost	Supplier D	702.6	1,510,505
Closest	Supplier A	69.7	1,529,565
Best Balanced Option	Supplier E	96.4	1,522,417.5
**Rebar**			
Lowest Cost	Supplier A	609.5	2367.27
Closest	Supplier C	56	2463.84
Best Balanced Option	Supplier C	56	2463.84
**Masonry**			
Lowest Cost	Supplier B	92	11,857.5
Closest	Supplier A	49.8	13,272.75
Best Balanced Option	Supplier B	92	11,857.5

**Table 9:** Results of performance testing of Multi-Origin GIS Routing Tool for different material classes.

Supplier Material	Number of Suppliers/Routes	Longest Routing Distance	Start Time (HH:MM:SS.HS)	End Time (HH:MM:SS.HS)	Approximate Routing Time (S.HS)
Cement	5	768.4	13:43:19.58	13:43:26.19	6.61
Structural Steel	5	702.6	13:57:40.13	13:57:48.29	8.16
Rebar	5	612.7	14:05:28.02	14:05:35.74	7.72
Masonry	5	552.3	14:21:45.33	14:21:48.61	3.28

**Table 10:** Comparison of GIS and webapp mapping tools to Multi-Origin GIS Router tool. (N/A means that specific methods are not directly capable of mutli-origin routing and related attributes to said routing are not applicable.)

Method	Webapp UI	Native Multi-Origin Routing	Does not Require Additional Plugins	Free to Use	Does Not Require Tuning or Domain Knowledge	User Is Not Required to Prepare Data	Specific Example of Studies Using the Tool
Google Maps (accessed September 2026) [64]	Yes	No	N/A	Yes	Yes	Yes	[7]
MapQuest (accessed September 2026) [65]	Yes	No	N/A	Yes	Yes	Yes	
Bing Maps (accessed September 2026) [66]	Yes	No	N/A	Yes	Yes	Yes	[30,41]
ArcGIS Desktop (Version 3.7) [62,67]	No	Yes, with plugin	Requires an additional plugin for multi-origin routing.	No	No	No (Requires layer data)	[1,2,4,5,8,13,15,16,19,20,21,23,24,25,28,31,32,33,35,36,37,46,47,48]
QGIS (Version 4) [63]	No	Yes	Requires additional plugin for addressing	No	No	No (Requires layer data)	[3,10,22,42]
UMMISCO GAMA (2025-06) [68]	No	Yes	Yes	Yes	No	No (Requires layer data)	[45]
Spoke (accessed September 2026) [59]	Yes	No, conceivably could be added via software development	N/A	Yes within small use cases	N/A	Yes	
Routific (accessed September 2026) [57]	Yes	No, conceivably could be added via software development	N/A	Yes, within small use cases	N/A	No	
Route4me (accessed September 2026) [58]	Yes	No, conceivably could be added via software development	N/A	No	N/A	Yes	
Multi-Origin GIS Router (Ours)	Yes	Yes	Yes	Yes	Yes	Yes	This study

## Data Availability

Complete data used for the case study may be provided upon reasonable request. Researchers and public institutions interested in using the Multi-Origin GIS Routing Tool should contact the authors at the coordinates provided.
